# Dual‐Mechanism Peptide SR25 has Broad Antimicrobial Activity and Potential Application for Healing Bacteria‐infected Diabetic Wounds

**DOI:** 10.1002/advs.202401793

**Published:** 2024-06-14

**Authors:** Xue‐Yue Luo, Chun‐Mei Hu, Qi Yin, Xiao‐Mei Zhang, Zhen‐Zhen Liu, Cheng‐Kai Zhou, Jian‐Gang Zhang, Wei Chen, Yong‐Jun Yang

**Affiliations:** ^1^ Department of Preventive Veterinary Medicine College of Veterinary Medicine Jilin University Changchun Jilin 130062 P. R. China

**Keywords:** antimicrobial peptides, diabetic wound, hydrogel, membrane disrupting, succinate:quinone reductase, uncultured bacteria

## Abstract

The rise of antibiotic resistance poses a significant public health crisis, particularly due to limited antimicrobial options for the treatment of infections with Gram‐negative pathogens. Here, an antimicrobial peptide (AMP) SR25 is characterized, which effectively kills both Gram‐negative and Gram‐positive bacteria through a unique dual‐targeting mechanism without detectable resistance. Meanwhile, an SR25‐functionalized hydrogel is developed for the efficient treatment of infected diabetic wounds. SR25 is obtained through genome mining from an uncultured bovine enteric actinomycete named *Nonomuraea Jilinensis* sp. nov. Investigations reveal that SR25 has two independent cellular targets, disrupting bacterial membrane integrity and restraining the activity of succinate:quinone oxidoreductase (SQR). In a diabetic mice wound infection model, the SR25‐incorporated hydrogel exhibits high efficacy against mixed infections of *Escherichia coli* (*E. coli*) and methicillin‐resistant *Staphylococcus aureus* (MRSA), accelerating wound healing. Overall, these findings demonstrate the therapeutic potential of SR25 and highlight the value of mining drugs with multiple mechanisms from uncultured animal commensals for combating challenging bacterial pathogens.

## Graphical abstract

The previously uncultured bacterium presents a viable strategy for the discovery of novel antimicrobial peptides. The antimicrobial peptide SR25 operates through a dual mechanism, targeting both the bacterial cell membrane and succinate:quinone oxidoreductase. It exhibits outstanding antibacterial efficacy and promotes the recovery of infected diabetes wounds.

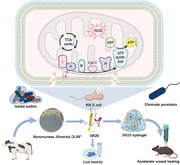

## 1 Introduction

Bacterial infections represent a major global cause of mortality, posing significant risks and imposing substantial healthcare burdens on public health.^[^ ^1^ ^]^ In diabetic individuals, hyperglycemia can lead to prolonged healing time in diabetic wounds due to microvascular dysfunction, hence considerably increasing susceptibility to bacterial infection.^[^ ^2^ ^]^ Although antibiotics conventionally serve as predominant pharmaceutical interventions against bacterial infections, their abuse has led to the formation of multidrug‐resistant bacteria, significantly impeding the effectiveness of antibiotic treatment.^[^ ^3^ ^]^ Consequently, there is an urgent need for potent antibacterial drugs and approaches capable of addressing bacterial infections and facilitating wound healing in diabetes patients while minimizing the risk of antibiotic resistance.

Antimicrobial peptides (AMPs) have shown significant promise as a novel class of therapeutic agents, yet the discovery of AMPs effective against Gram‐negative organisms remains challenging due to their inherent penetration barriers.^[^ ^4^ ^]^ The prevailing consensus posits that AMPs primarily target cell membranes through unique mechanisms, leading to membrane perturbation and the disruption of associated physiological events.^[^ ^5^ ^]^ This disruption has traditionally been believed to make the development of AMP resistance highly improbable.^[^ ^6^ ^]^ Regrettably, recent investigations have revealed instances of resistance to these peptides, at least in vitro.^[^ ^7^ ^]^ Consequently, there exists a pressing need to develop innovative AMPs with alternative killing mechanisms. Through in‐depth exploration in microbiome research, numerous innovative therapeutic agents have been unearthed. Recently identified AMPs derived from nematode genome data, human gut microbiome data and rumen microbiome metagenome data emphasize the potential for administering bioinformatics‐based drugs to effectively address bacterial infections.^[^ ^8^ ^]^ A comprehensive understanding of the genome holds the key to identifying and harnessing the beneficial features embedded within these genetic structures.

Herein, we isolated a previously uncultured actinomycete, *Nonomuraea Jilinensis* DL99^T^, from the intestinal tract of dairy cows. Through genome data mining, we acquired the peptide SR25, which exhibits broad antimicrobial activity. Utilizing the collected data, we propose a reasonable dual target mechanism of SR25. Inspiringly, recent research has underscored the favorable attributes of hydrogel dressings as effective delivery systems for small molecule drugs. These dressings successfully maintain the biological activity of loaded small molecule peptides while also creating a moist, favorable environment for the in vivo repair of nearby wounded tissues.^[^ ^9^ ^]^ Consequently, SR25 was incorporated into the hydrogel matrix, leading to the significant inhibition of bacterial infection and an expedited healing process in the wounds of diabetic mice. In summary, SR25 represents a promising antibacterial agent, encouraging its use as a natural antimicrobial agent for treating infected diabetic wounds.

## 2 Results

### 2.1 Identification and Genomic Characteristics of *Nonomuraea Jilinensis* sp.nov. 

**Figure 1:**
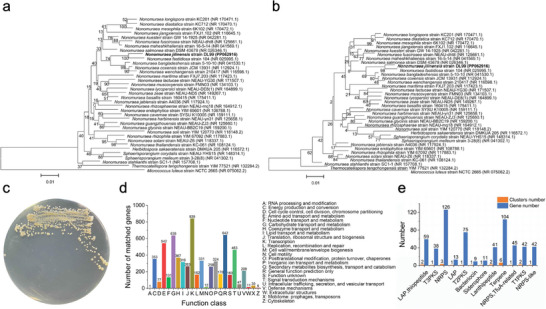
Evolutionary relationships and whole genome analysis of *Nonomuraea Jilinensis* sp. nov. a,b) Phylogenetic trees inferred by the NJ a) and ML b) methods based on 16S rRNA gene sequences showing the relationships between *Nonomuraea Jilinensis* DL99^T^ (in bold) and the most closely related organisms from the family Streptosporangiaceae. The strain DL99^T^ was most closely related to *Nonomuraea fastidiosa* 104^T^, *Nonomuraea coxensis* JCM 13931^T^, and *Nonomuraea wenchangensis* 210417^T^ with 16S rRNA gene sequence similarities of 98.17%, 97.73% and 97.31%, respectively. The 16S rRNA gene sequence of *Micrococcus luteus* strain NCTC 2665^T^ was used as an outgroup. Bootstrap values (percentages of 1000 replications) are shown next to the branches. The scale represents 0.01 nucleotide substitutions per site. c) Conlony morphology of *Nonomuraea Jilinensis* DL99^T^ aerobic culture on the ISP2 agar plate. d) COG functional gene classification. e) Secondary metabolic gene clusters and the number of corresponding genes.

Uncultured bacteria provide an abundant source of novel natural products. Herein, for the first time, we isolated strain DL99^T^ from the intestinal tract of dairy cows. Phylogenetic analysis revealed the distinct branch of strain DL99^T^ in the Nonomuraea genus, in which the similarity of 16S rRNA sequence was less than the recommended thresholds (98.65% for species) (**Figure** **1**a,b).^[^ ^10^ ^]^ On ISP2 (International Streptomyces Project 2 medium) agar, the strain DL99^T^ displayed circular, raised, light‐yellow colonies (1‐2 mm diameter) with Gram‐positive staining and wavy sporulating hyphae (Figure 1c; Figure S1a, Supporting Information). Compared to *Nonomuraea fastidiosa* 104^T^,^[^ ^11^ ^]^ the closest strain to which physiological data are available, the strain DL99^T^ demonstrated a broader pH tolerance (4.0–10.0) and NaCl concentration range (0–5.0), but lacked the ability to hydrolyze gelatin (Table S1, Supporting Information). Furthermore, the digital DNA–DNA hybridization (dDDH) and average nucleotide identity (ANI) values (0.23% to 30%, 76.42% to 87.48%, respectively) with reference species confirmed DL99^T^’s differentiation below the proposed thresholds (70%, 95%) (Table S2, Supporting Information).^[^ ^12^ ^]^ In conclusion, the strain DL99^T^ is distinguishable from closely related Nonomuraea species. We suggest *Nonomuraea Jilinensis* sp. nov. as a new species in the Nonomuraea genus, with strain DL99^T^ serving as the type strain. Under accession number PP062916, the 16S rRNA gene sequence of DL99^T^ has been uploaded to GenBank.

A circular chromosome containing 8586,945 bps comprised the complete genome of strain DL99^T^ (Figure S1b, Supporting Information). Functional gene annotation revealed 170 genes associated with secondary metabolite biosynthesis, transport, and catabolism (Figure 1d). Further analysis of the secondary metabolic gene cluster revealed abundant secondary metabolites (Figure 1e). These results underscored the potential of the uncultured strain DL99^T^ for the identification of novel natural products.

### 2.2 Prediction and Characterization of Antimicrobial Peptides

**Figure 2:**
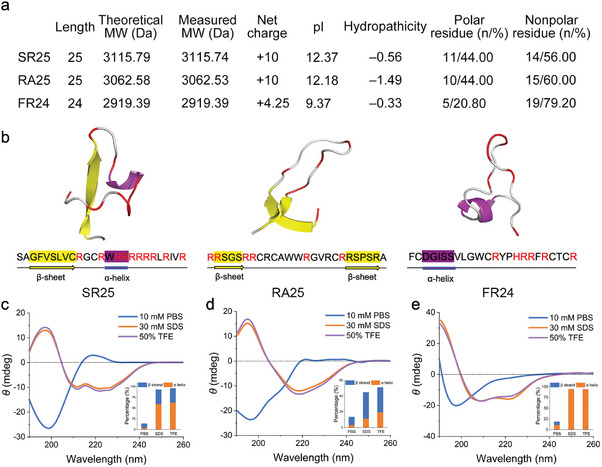
Physico‐chemical properties and structure of the peptides. a) Physico‐chemical properties of peptides. b) 3D ribbon models of peptides predicted by I‐TASSER and visualized using PyMol software. The α‐helix is covered with purple and the β‐sheet is covered with yellow. The positively charged residues in sequence are marked as red. c–e) CD spectra of SR25 c), RA25 d), and FR24 e) in different environments. The α‐helix and β‐sheet contents (%) of these peptides were inserted. Spectra is the accumulation of three scans carried out with 100 µmol L^−1^ peptide in 10 m_M_ PBS, 10 m_M_ SDS in PBS or 50% TFE in PBS. MWs, molecular weights; pI, isoelectric point.

To predict novel and promising AMPs, we conducted an analysis of all the open reading frames (ORFs) less than 200 bps in the DL99^T^ genome, screening for sequences with helical or disulfide bonds, positive net charge, protein binding potential > 0 kcal mol^−1^, and peptide formation rate > 0.9. Finally, we selected the three peptides with the highest probability of having low MIC scores in HydrAMP as potential active AMPs. The sequences of the three peptides exhibited no significant similarities to any peptide in the CAMPR3 or APD3 database, confirming their novelty (Table S3). The physico‐chemical characteristics of the promising AMPs were identified by online software (**Figure** **2**a). To confirm that the synthesized peptides were consistent with the desired specifications, the peptides were characterized by reversed‐phase high‐performance liquid chromatography (RP‐HPLC) and electrospray ionization mass spectrometry (ESI‐MS) (Figure S2, Supporting Information). The purities of the peptides were higher than 95%, and there was little difference between the actual and expected molecular weights. (Figure 2a). The structures of the peptides were predicted using I‐TASSER (Figure 2b). Subsequently, the secondary structures of the peptides were inspected via circular dichroism (CD) spectroscopy. In environments mimicking the hydrophobic conditions of microbial membranes and the anionic milieu of phospholipid bilayers (50% trifluoroethanol solution and 10% sodium dodecyl sulfate micelles), RA25 displayed a typical α‐helix structure (Figure 2d), while FR24 exhibited a β‐sheet structure (Figure 2e). The CD spectrum of SR25 demonstrated both characteristics (Figure 2c), with a relatively higher content of α‐helix than β‐sheet, as indicated by the inserted figure in the CD spectrum. These findings collectively validated that the AMPs we screened and synthesized aligned with the predicted information.

### 2.3 Antimicrobial Efficacy and Biocompatibility Evaluation

**Figure 3:**
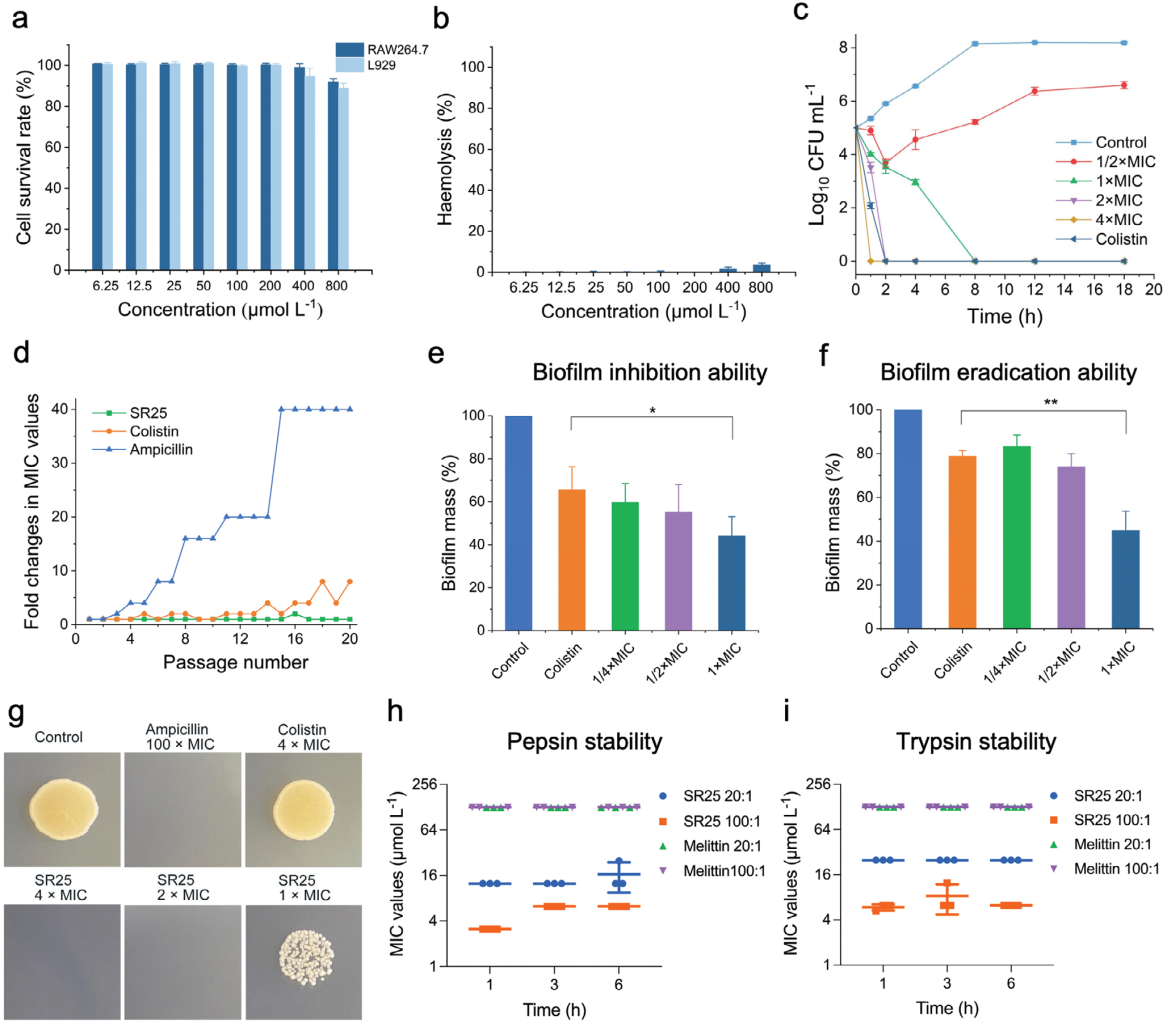
Antibacterial efficacy and biosafety of SR25 in vitro. a) Cytotoxicity of SR25 was evaluated using CCK‐8 assay on Raw 264.7 cell line and L929 cell line. b) Haemolytic activity of SR25 to the RBCs of C57BL/6 mice. PBS was used as the negative control, 1.0% Triton X‐100 was used as the positive control. c) Time‐kill kinetics curve of *E. coli* after exposure to SR25, colistin at 4 × MIC was used as the positive control, *E. coli* without treatment was used as the control. d) Induced resistance assay of SR25, colistin, and ampicillin against *E. coli* for a continuous passage of 20 cycles. e) Effect of SR25 on biofilm formation in *E. coli*, biofilm without treatment as the control, colistin at 4 × MIC was used as the positive control. f) Effect of SR25 on biofilm eradication in *E. coli*, biofilm without treatment as the control, colistin (4 × MIC) was used as a positive control. g) Bactericidal activity of SR25 against persisters induced from *E. coli*. Rifampicin (100 µg mL^−1^) was used to induce the formation of persisters, ampicillin (90 µ_M_, 10 × MIC) was used to eliminate non‐persisters, persisters without treatment as the control, ampicillin (900 µmol L^−1^, 100 × MIC) and colistin (25 µmol L^−1^, 4 × MIC) were used as positive controls. h,i) Protease sensitivity of SR25. The MIC values of *E. coli* incubated with the SR25 after the treatment of pepsin h) or trypsin i), linear peptide melittin were used as the control. Three biologically separate experiments were carried out, data presented as mean ± SD, *n* = 3, ^*^ *p *< 0.05, ^**^ *p* < 0.01.

SR25 exhibited potent broad‐spectrum antibacterial activity, inclusive of drug‐resistant bacteria, with the MICs (minimum inhibitory concentrations) ranging from 1.57 to 12.5 µmol L^−1^ against the tested strains (**Table** **1**). Notably, the antibacterial activity of SR25 against *E. coli* was matched that of colistin, which is thought to be the final line of defense against Gram‐negative infections. Among the three candidate peptides, SR25 demonstrated minimal cytotoxicity (12%) and hemolysis rate (3.6%) even at a relatively high concentration (800 µmol L^−1^) (**Figure** **3**a,b; Figure S3a–d, Supporting Information). These findings highlighted the remarkable biocompatibility and high selectivity of SR25 for bacteria, warranting its selection as the preferred AMP for subsequent studies.

**Table 1:** Antibacterial activities of the peptides.

	MIC [µmol L^−1^]				
Species	SR25	RA25	FR24	Amp^a)^	Col^b)^
*Salmonella typhimurium* SL1344	3.13	>100	>100	12.5	3.13
*Klebsiella pneumoniae* K7	>100	>100	>100	>71.6	6.25
*Escherichia coli* O157:H7	3.13	>100	>100	12.5	3.13
*Pseudomonas aeruginosa* ATCC 27853	6.25	>100	>100	>71.6	6.25
*Bacillus subtilis* BL‐4	3.13	>100	>100	1.57	6.25
*Salmonella pullorum* ATCC19945	6.25	25	>100	35.8	18.5
Methicillin‐Resistant *Staphylococcus aureus* USA300	6.25	16	>100	71.6	18.5
*Staphylococcus aureus* ATCC25904	12.5	6.25	>100	>71.6	>18.5
*Staphylococcus aureus* ATCC25923	12.5	6.25	>100	1.57	>18.5
*Staphylococcus aureus* ATCC29213	12.5	6.25	>100	1.57	>18.5
*Enterococcus faecalis* 4P‐SA	25	6.25	>100	>71.6	>18.5
*Enterococcus faecalis* ATCC51299	12.5	12.5	>100	12.5	>18.5
*Listeria monocytogenes* EGD	3.13	6.25	>100	4.5	>18.5
*Listeria monocytogenes* 10403S	3.13	3.13	>100	25	>18.5
*Listeria monocytogenes* ATCC19115	3.13	3.13	12.5	6.25	>18.5
*Staphylococcus epidermidis* YYF‐771	1.57	1.57	>100	1.57	9.2

^a)^ Amp, Ampicillin;

^b)^ Col, Colistin.

To further evaluate the antimicrobial potency of SR25, a killing kinetic assay was performed, and the results revealed that SR25 at 4 × MIC eradicated all *E. coli* cells within 1 h, surpassing colistin (4 × MIC), which required 2 h for comparable effects (Figure 3c). Sub‐MIC treatment with SR25 for 20 passages did not induce resistance in *E. coli* and MRSA (Figure 3d, Figure S3e, Supporting Information). Moreover, at a low concentration (1 × MIC, 3.13 µmol L^−1^), SR25 disrupted *E. coli* biofilm formation (55%), outperforming colistin (35%) (Figure 3e). At the same dose, SR25 eliminated 56% of the biofilm, which was significantly greater than that of colistin (22%) (Figure 3f). Persisters are a subpopulation of bacterial cells that can enter a dormant state and tolerate antimicrobial therapy. These cells have emerged as key players in the recurrence of biofilm infections. Our results showed that SR25 at 2 × MIC completely eradicated persisters, whereas colistin at 4 × MIC had little effect on the survival *E. coli* (Figure 3g). We further investigated the stability of SR25. Extreme pH and high‐temperature environments did no influence on the activity of SR25 (Figure S3f,g, Supporting Information). After incubating with trypsin or pepsin, SR25 exhibited relatively stable activity changes compared to melittin, another linear peptide (Figure 3h,i). This stability is attributed to the ability of SR25 to maintain a stable secondary structure (Figure S3h,i, Supporting Information). Collectively, these findings established that SR25 possessed the capability to act as a highly efficient antimicrobial agent.

### 2.4 Preliminary Action Mechanism of SR25

**Figure 4:**
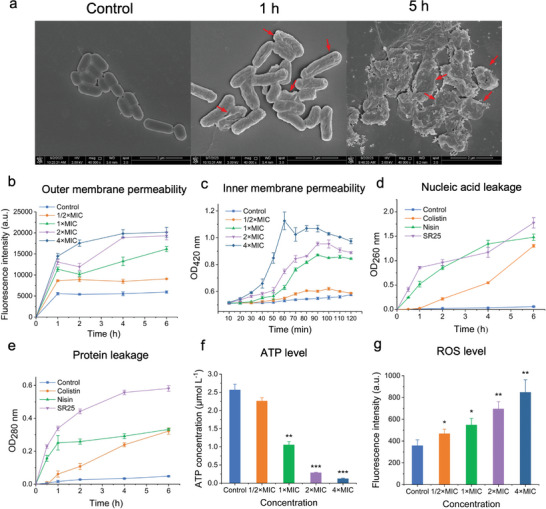
Membrane system as a preliminary target. a) SEM photomicrographs of *E. coli* treated with SR25, *E. coli* without treatment was used as the control. Scale bar = 2 µm. Red arrows show the damaged cell membranes. The images presented are indicative of three biologically distinct tests conducted with similar results. b) Outer membrane permeability of *E. coli* treated with different concentrations of SR25 determined by NPN assay. c) Inner membrane permeability of *E. coli* treated with different concentrations of SR25 indicated by the hydrolysis of ONPG. d,e) Leakage of cellular contents. Measurement of cellular leakage of nucleic acid d) and protein e) from *E. coli* after SR25 (1 × MIC) treatment for different durations. Colistin and nisin were used as positive controls, untreated *E. coli* was used as the control. f) Level of intracellular ATP in *E. coli* treated with SR25, untreated *E. coli* was used as the control. g) ROS generation in *E. coli* treated with SR25, untreated *E. coli* was used as a control. Three biologically separate experiments were carried out, data presented as mean ± SD, *n* = 3, ^*^ *p *< 0.05, ^**^ *p* < 0.01, ^***^ *p* < 0.001; SEM, scanning electron microscopy; OD, optical density; ONPG, O‐nitrophenyl‐beta‐D‐galactopyranoside; MIC, minimum inhibitory concentration; ATP, adenosine triphosphate; ROS, reactive oxygen species.

Based on the cationic and hydrophobic physicochemical properties of SR25, it is reasonable to hypothesize that SR25 operates on the membrane similarly to other cationic peptides. To directly visualize the impact of SR25 treatment on bacterial cells, scanning electron microscopy (SEM) was employed to discern morphological changes. Untreated *E. coli* cells displayed long rod shapes with intact and smooth surfaces. Following exposure to SR25, the cell surfaces exhibited increased roughness and crenation. Prolonged treatment led to severe surface damage, resulting in the loss of cellular morphology and accompanied by content leakage (**Figure** **4**a). Meanwhile, we found that SR25 considerably enhanced the permeability of *E. coli* outer membrane (OM) (Figure 4b), and administration of SR25 led to a fast enhancement in the inner membrane (IM) permeability within 90 min (Figure 4c). Moreover, we observed that SR25 caused nucleic acid and protein leakage from within the cell (Figure 4d,e). These results were similar to the membrane function of colistin (Figure S4a,b, Supporting Information). Interestingly, the intracellular adenosine triphosphate (ATP) content decreased significantly (Figure 4f), and the amount of reactive oxygen species (ROS) released increased significantly (Figure 4g). Plausible explanations for these results include ATP leakage resulting from membrane damage or SR25 interacting with intracellular targets post‐membrane penetration, influencing energy metabolism and intracellular respiration, ultimately inducing oxidative stress. Regardless, based on the aforementioned findings, a more detailed investigation into the mechanism of SR25 in *E. coli* seems warranted.

### 2.5 Multi‐Omics Investigation of Further Action Mechanism

**Figure 5:**
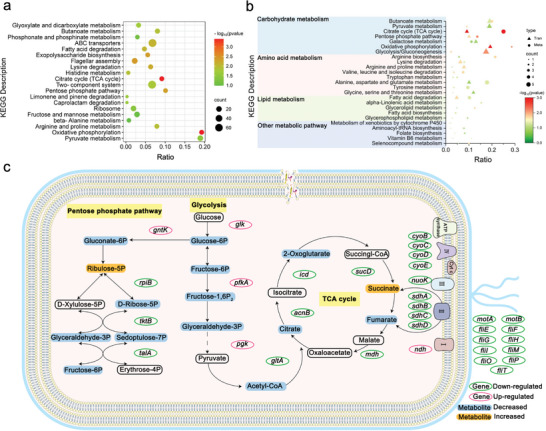
Further exploration of the mechanism of SR25. a) Analysis of KEGG pathway enrichment on differentially expressed genes between SR25‐treated and untreated group. Plotting of significantly enriched pathways was chosen (*p* < 0.05). b) KEGG pathway enrichment analysis between SR25‐treated and untreated group using integrated transcriptomic and metabolomic data. The top 25 highly‐enriched pathways are shown. c) Hypothetical route by integrated gene‐by‐metabolite interactions. *gntK*, D‐gluconate kinase; *rpiB*, ribose‐5‐phosphate isomerase B; *tktB*, transketolase 2; *talA*, transaldolase A; *glk*, glucokinase; *pgk*, phosphoglycerate kinase; *icd*, isocitrate dehydrogenase; *sucD*, succinyl‐CoA synthetase subunit alpha; *mdh*, malate dehydrogenase; *acnB*, aconitate hydratase 2/2‐methylisocitrate dehydratase; *gltA*, citrate synthase; *cyoB*, cytochrome bo3 subunit 1; *cyoC*, cytochrome bo3 subunit 3; *cyoD*, cytochrome bo3 subunit 4; *cyoE*, heme O synthase; *sdhA*, succinate:quinone oxidoreductase (FAD binding protein); *sdhB*, succinate:quinone oxidoreductase (iron‐sulfur cluster binding protein); *sdhC*, succinate:quinone oxidoreductase (membrane protein SdhC); *sdhD*, succinate:quinone oxidoreductase (membrane protein SdhD); *ndh*, NADH:quinone oxidoreductase II; *motA*, motility protein A; *motB*, motility protein B; *fliE*, flagellar protein FliE; *fliF*, flagellar basal‐body MS‐ring and collar protein; *fliG*, flagellar motor switch protein FliG; *fliH*, flagellar biosynthesis protein FliH; *fliI*, flagellar export ATPase FliI; *fliM*, lagellar motor switch protein FliM; *fliO*, flagellar biosynthesis protein FliO; *fliP*, flagellar biosynthesis protein FliP; *fliT*, flagellar biosynthesis protein FliT; I, respiratory complex I; II, respiratory complex II; III, respiratory complex III; IV, respiratory complex IV; Cyt c, cytochrome c; ATP, adenosine triphosphate.

To decipher the action mechanisms of SR25, we performed transcriptomic and metabolomic analyses of *E. coli* exposed to SR25 (1 × MIC) for 1 h. The treated group exhibited 1158 differentially expressed genes (DEGs) (414 upregulated, 744 downregulated) and 395 differentially expressed metabolites (DEMs) (276 upregulated, 119 downregulated) compared to the untreated groups (Figure S5a,b, Supporting Information). The credibility of the transcriptomic data was confirmed by qRT‒PCR verification (Figure S5c,d, Supporting Information). Aligned with the observed membrane action mechanism, a large amount of DEGs were enriched in the “ABC transporters” and the “two‐component system” pathways (**Figure** **5**a). Integrated transcriptomic and metabolomic data revealed 25 highly enriched pathways (Figure 5b), unveiling key aspects of the inactivation mechanism (Figure 5c): 1) inhibition of the tricarboxylic acid cycle (TCA cycle) and oxidative phosphorylation pathway; 2) upregulation of the glycolysis pathway and disturbance of the pentose phosphate pathway (PPP).

The KEGG enrichment pathway of the joint analysis indicated significant changes in the TCA cycle and oxidative phosphorylation pathway (Figure 5b; Tables S4,  S5, Supporting Information). The key TCA cycle genes (*acnB*, *sucD*, *sdhABCD*, and *icd*) and the metabolites (acetyl‐CoA, citric acid, α‐ketoglutaric acid, and fumaric acid) were downregulated, suggesting that the TCA cycle was inhibited. Remarkably, we noted an increase in the content of succinic acid, which is converted into fumaric acid through the catalysis of respiratory complex II. This observation prompted an investigation into complex II, a pivotal enzyme in the TCA cycle and a crucial player in the respiratory chain. Correspondingly, the expression of genes related to complex II (*sdhABCD*), complex III (*nuoK*), and complex IV (*cyoB*, *cyoC*, *cyoD*, and *cyoE*) was significantly downregulated in the oxidative phosphorylation pathway, suggesting electron transport chain blockage and likely decreased ATP synthesis, consistent with the observed decrease in the intracellular ATP concentration.

SR25 treatment also significantly disturbed glycolysis and the PPP. We observed the upregulation of *glk*, *pfkA*, and *pgk* in the glycolysis pathway, accompanied by the increased consumption of the metabolites. Concurrently, D‐ribose‐5‐phosphate and D‐sedoheptulose‐7‐phosphate were depleted. The disturbance in the PPP was further indicated by the upregulation of *gntK* and the significant downregulation of *rpiB*, *tktB*, and *talA*. This perturbation aligns with microbial responses to environmental pressures, resulting in heightened energy demands and augmented glycolytic activities.^[^ ^13^ ^]^ Overall, the intricate impact of SR25 on energy metabolism suggests a potential inactivation mechanism. To delve into this mechanism, we performed a series of tests to investigate the distinctive features of complex II as an energy metabolism target.

### 2.6 Identification of Interaction Mechanism between SR25 and Complex II

**Figure 6:**
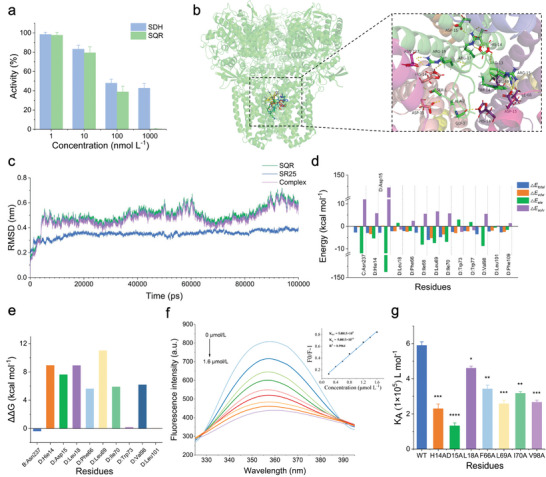
Interaction mechanism between SR25 and SQR. a) Inhibition of SQR and SDH activities by SR25 (*n* = 3). b) Molecular docking of SR25 with SQR (2WDV). c) RMSD analysis of 100 ns trajectory for the SR25‐SQR complex. d) Decomposition of the MM/PBSA binding energy on a per‐residues in the SR25‐SQR complex. e) Computational alanine scanning mutagenesis data for the SR25‐SQR complex. *ΔΔG* = *ΔG*_MT_‐*ΔG*_WT_. f) Emission spectra of purified protein SdhD at different concentrations of SR25, the insert shows the Stern–Volmer plots for the quenching of SDHD by SR25. Kq is the quenching rate constant of the bimolecule, K*_SV_* stands for the Stern–Volmer dynamic quenching constant. g) Binding constants (K_A_) of SR25 with SDHD and its mutants (*n* = 3). ^*^ *p *< 0.05, ^**^ *p* < 0.01, ^***^ *p* < 0.001, ^****^ *p* < 0.0001; SDH, succinate dehydrogenase; SQR, succinate:quinone oxidoreductase; RMSD, root mean square deviation; *E_total_*, total energy; *E_vdw_*, van der Waals energy, *E_ele_*, electrostatic energy; *E_solv_*, solvation free energy; ΔΔG, change in the Gibbs free energy upon mutation to alanine; SdhD, membrane protein SdhD of succinate:quinone oxidoreductase; *K*_A_, binding constants; MT, mutant type; WT, wild type.

To determine the impact of SR25 on complex II, we performed a panel of experiments. In complex II, the succinate dehydrogenase (SDH) activity constitutes a partial reaction of SQR.^[^ ^14^ ^]^ Notably, SR25 completely inhibited the SQR activity of complex II from *E. coli*; however, complete inhibition of SDH activity was not achieved even at 1 000 nmol L^−1^ (**Figure** **6**a). This partial inhibition may result from the inability of SR25 to entirely prevent electron transfer from iron‐sulfur centers to water‐soluble dyes via a nonphysiological pathway.^[^ ^14a^ ^]^ Subsequently, we applied a molecular docking strategy to observe the binding mode of SR25 with SQR (Figure 6b; Table S6, Supporting Information). The root mean square deviation (RMSD) reached a steady state of ≈0.5 nm after 4 ns, resulting in a stable docking trajectory (Figure 6c). Extremely, we noticed that a specific group of residues played a significant role in determining the binding affinity (Figure 6d), with electrostatic interactions and van der Waals forces contributing substantially to the binding energy (Table S7, Supporting Information). Positions that exhibit a major change in binding energy (>1.5 kcal mol^−1^) when mutated to alanine are additionally referred to as “hot spots”.^[^ ^15^ ^]^ We further found that only the SdhD residues (Hie14, Asp15, Leu18, Phe66, Leu69, Ile70 and Val98) as hot spots for the SR25‐SQR interaction system (Figure 6e). Subsequently, we purified the SdhD protein from *E. coli* and the alanine mutants of major amino acids to verify these findings. Consistently, we confirmed the binding of SR25 to SdhD by recording the fluorescence spectra. The *Kq* value (5.0813 × 10^13^ L mol^−1^ s^−1^) was higher than the limiting diffusion constant (2 × 10^10^ L mol^−1^ s^−1^), which suggested that the acceptable quenching mechanism is a static quenching process associated with the emergence of the SR25‐SdhD complex (Figure 6f). Moreover, the binding constants for all the mutants were significantly lower than that of wild‐type SdhD (Figure 6g), underscoring the crucial role of these residues in the binding of SR25 to SdhD. Collectively, our results affirmed the predominant binding of SR25 to the SdhD subunit of SQR, resulting in the inhibition of its activity. This established SQR as a distinct target for SR25, setting it apart from previously reported AMPs. In addition, membrane‐related experiments and enzyme activity detection were conducted to evaluate the impact of SR25 on various other Gram‐negative and Gram‐positive pathogens, and as expected, SR25 demonstrated varying degrees of damage to membrane permeability and SQR activity (Figure S6, Supporting Information). In summary, these results indicated that SR25 exerted its broad‐spectrum antibacterial activity through a unique dual mechanism.

### 2.7 Therapeutic Efficacy of SR25 Hydrogel in Infected Diabetic Wounds

**Figure 7:**
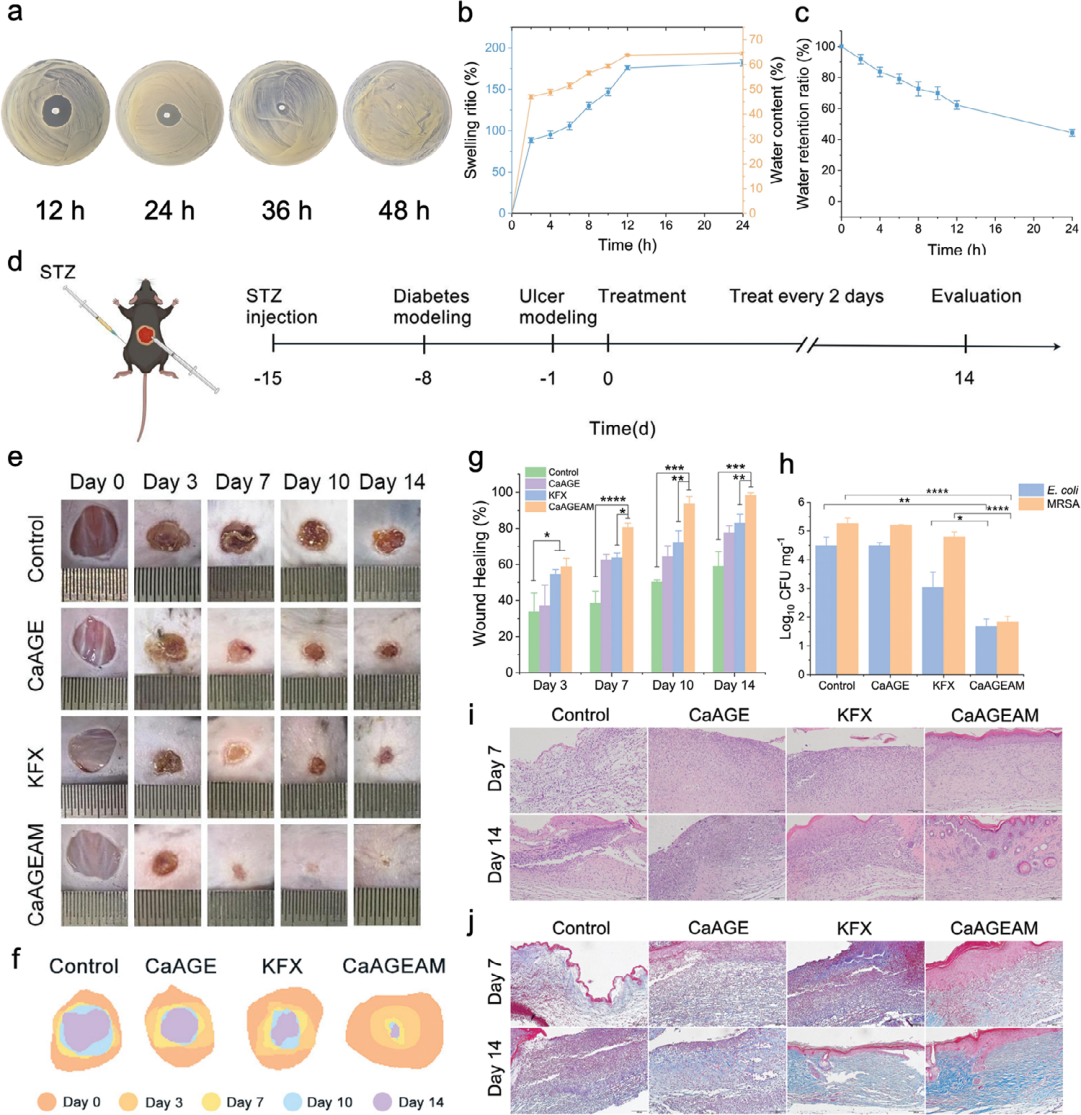
Therapeutic potential of SR25 in a mouse model of infected diabetic wounds. a) Antibacterial property of CaAGEAM evaluated by the inhibition zone (*n* = 3). b) Swelling rate and water content of CaAGEAM in PBS for 24 h (*n* = 3). c) Water retention ratio of CaAGEAM (*n* = 3). d) Experimental scheme for establishing the mouse model of bacteria‐infected diabetic wound healing. e) Representative images of wound changes in diabetic mice after treatment. f) Traces of wound closure in diabetic mice after treatment. g) Wound healing percentage at different time points (day 3, day 7, day 10, and day 14) (*n* = 3). h) Colony counts of *E. coli* and MRSA remaining in wounds of diabetic mice after treatments (*n* = 6). i) Representative images of H&E staining of diabetic wound tissue on different days to measure epithelialization. j) Representative images of MT staining of diabetic wound tissue on different days to measure collagen deposition. PBS treatment group as the control, CaAGE represents sodium alginate/Ca^2+^/gelatin gels, KFX represents Kangfuxin, CaAGEAM represents sodium alginate/Ca^2+^/gelatin/antimicrobial peptide SR25. ^*^ *p* < 0.05, ^**^ *p* < 0.01, ^***^ *p* < 0.001, ^****^ *p* < 0.0001; STZ, streptozotocin; MRSA, methicillin‐resistant *Staphylococcus aureus*.

First, the CaAGEAM hydrogel, which consisted of sodium alginate, Ca^2+^, gelatin, and the antimicrobial peptide SR25, was prepared using a simple in‐situ release process. We evaluated the antibacterial sustained release effect of the CaAGEAM hydrogel. The inhibition zone was observed at 12 h (24.07 ± 1.36 mm), 24 h (17.40 ± 0.62 mm) and 36 h (10.03 ± 1.14 mm) (**Figure** **7**a). Meanwhile, we observed that the release rate of SR25 was the fastest at 0–8 h and the release rate reached 70.88% at 24 h (Figure S7a, Supporting Information). Moreover, the swelling rate of the CaAGEAM hydrogel at 24 h was 182.08%, and the change in water content followed a trend similar to that of the swelling rate (Figure 7b). Satisfactorily, the water retention rate of the hydrogel was 91.80% at 2 h, and after a 24 h period at room temperature, the hydrogel retained ≈50% of its initial water content (Figure 7c). Furthermore, the CaAGEAM hydrogel exhibited no cytotoxicity or hemolytic activity toward mammalian cells (Figure S7b,c, Supporting Information). Collectively, these findings highlighted the potential of the CaAGEAM hydrogel as a wound dressing.

Next, to evaluate the therapeutic efficacy of SR25, we constructed a model of wound infection in diabetic mice (Figure 7d). Blood glucose levels in diabetic mice were continuously monitored during the wound healing period (Figure S7d, Supporting Information). First, photographs of the wounds and quantification of the closure rates on days 3, 7, 10, and 14 following the trauma demonstrated that the CaAGEAM hydrogel notably accelerated diabetic wound healing (Figure 7e–g). Notably, the wound healing rate reached 93.69% on postoperative day 10, surpassing the efficacy of KFX (72.17%), a commonly employed clinical treatment (Figure 7g). Second, compared with those in the other groups, the number of colonies of both MRSA (1.8‐log) and *E. coli* (1.7‐log) was significantly lower in the CaAGEAM hydrogel group (Figure 7h). Third, hematoxylin and eosin (H&E) staining revealed that the CaAGEAM hydrogel group displayed a nearly normal histological status (Figure 7i), with increased production of skin appendages such as new blood vessels and hair follicles. Fourth, according to the Masson's trichrome (MT) staining, the CaAGEAM group exhibited well‐arranged fibers (Figure 7j), with significantly higher collagen density than other groups (Figure S7e, Supporting Information). Finally, examination of heart, liver, spleen, lung, and kidney tissues from all treatment groups did not exhibit any evident pathological damage (Figure S7f, Supporting Information). In conclusion, the hydrogels containing SR25 exhibited antibacterial properties, facilitated re‐epithelialization, and promoted collagen deposition at the wound site. These effects collectively accelerated skin regeneration, offering a promising strategy for the complete healing of full‐thickness diabetic wounds.

## 3 Discussion

Conventional screening methods have been unsuccessful in discovering new antibiotics in recent years.^[^ ^16^ ^]^ Traditional screening sources seem overexploited and tend to produce compounds that are already known. However, uncultured bacteria represent a significant and untapped reservoir of novel natural product frameworks.^[^ ^17^ ^]^ This potential has been substantiated by Rhythm et al., whose research established the efficacy of clovibactin, derived from uncultured soil bacteria, in effectively controlling drug‐resistant Gram‐positive bacterial infections.^[^ ^18^ ^]^ In the context of this study, we identified an uncultured actinomycete species, designated as *Nonomuraea Jilinensis* sp. nov. This novel species emerges as a prolific source of secondary metabolites, contributing to the expanding landscape of antibiotic discovery.

For several decades, the primary method for identifying natural compounds was isolation based on bioactivity. Although the success rate of this approach for identifying bioactive natural compounds was quite high, it also presented a unique set of problems. The process of identifying and isolating an active component from a complex mixture can be excessively time‐consuming. Moreover, this untargeted method frequently leads to the repeated isolation of the same chemicals.^[^ ^19^ ^]^ To find new natural compounds, researchers are increasingly depending on genome mining techniques.^[^ ^20^ ^]^ Indeed, the data obtained from single‐cell genomics research and metagenome sequencing have expanded the possibilities for isolating specific natural products by discovering customized tactics that leverage the particular characteristics of the target species to achieve efficient isolation. These techniques have great potential for using the abundant genetic and biological resources that uncultivated microorganisms offer.^[^ ^21^ ^]^ SR25, AMSIN,^[^ ^22^ ^]^ HG2, and HG4^[^ ^8b^ ^]^ are both successful examples of this method. Differently, SR25 not only is effective against drug‐resistant Gram‐positive bacteria but also quickly kills Gram‐negative bacteria.

Cationic AMPs are generally considered to engage with negatively charged bacterial membranes, thereby manifesting their antibacterial activity.^[^ ^23^ ^]^ However, the findings presented in this study offer a distinct perspective. Our results indicate that SR25 inhibits the activity of SQR along with membrane penetration and pore formation. Indeed, SQR has long been recognized as a pivotal target for the development of pharmaceuticals and agrochemicals.^[^ ^24^ ^]^ Regrettably, recent reports highlight that point mutations in the SdhB and SdhC subunits might confer resistance to pydiflumetofen.^[^ ^25^ ^]^ The exceptional rarity of resistance to SR25 may be attributed to its dual targets. Consequently, we propose SR25 as a potential template for dual‐mechanism antibiotics, offering a strategic approach to counteract the escalating antibiotic resistance crisis. Additionally, our DEGs analysis revealed a significant downregulation of genes associated with flagellar assembly and movement (Figure 5a,c; Table S4, Supporting Information). These observations suggested that SR25 affected the flagella system in *E. coli*. However, additional investigations are imperative to elucidate whether the effect represents an inhibitory impact on bacterial movement or a survival strategy employed by bacteria to mitigate environmental stress. The nuanced understanding of the multifaceted interactions of SR25 with bacterial targets, as revealed in this study, holds promise for advancing our comprehension of antibiotic action and resistance mitigation strategies.

A significant concern in diabetic wound infections is the complexity of mixed bacterial infections, contributing to prolonged wound healing times, extended periods of inflammation, and a heightened risk of ulcer recurrence.^[^ ^26^ ^]^ Naturally, an ointment that eliminates these microorganisms holds enormous promise for treating diabetes patients who have recurring infections and preventing the development of antibiotic resistance and transmission. According to earlier research, this CaAGE hydrogel basis was ideal for the AMPs.^[^ ^27^ ^]^ in terms of the stability, release profile, and antibacterial activity of the peptide. Notably, our research substantiated the success of SR25‐containing hydrogel in effectively eliminating both *E. coli* and MRSA within an established diabetic wound infection model. In recent investigations, various AMPs, including cecropin,^[^ ^27b^ ^]^ SCIBIOIII,^[^ ^28^ ^]^ QHREDGS,^[^ ^29^ ^]^ and LL‐37,^[^ ^30^ ^]^ have been researched for their antimicrobial activity and healing rate in diabetic wound infections. However, none of these peptides demonstrated the capacity to significantly reduce bacterial counts by ≈3‐log or expedite wound healing within a 10‐day timeframe. These findings are consistent with the peptide's effectiveness profile, suggesting that topical administration of the SR25 hydrogel is a promising treatment for removing mixed bacteria in wounds and accelerating wound healing; this treatment could even be applied to burn wounds and atopic dermatitis lesions.

The primary weakness of this study is that the efficacy trials were limited to full‐thickness wounds. In deeper wounds, proteolytic enzymes, wound debris, and/or inadequate penetration to deeper layers can all hinder the effectiveness of treatments. Personalized drug delivery methods, such as encapsulated and controlled‐release nanoparticles, can help overcome these obstacles.^[^ ^31^ ^]^ Another noteworthy constraint pertains to the exploration of whether SR25 assumes additional roles in expediting wound healing under diabetic conditions, specifically in fostering cell proliferation, migration, and angiogenesis. However, it is imperative to underscore that further investigative endeavors are requisite to validate and substantiate these preliminary findings.

In summary, we successfully identified a novel AMP sourced from *Nonomuraea Jilinensis*, denoted as SR25. Significantly, SR25 operated through a unique mechanism to effectively eradicate Gram‐negative bacteria while simultaneously inhibiting biofilm formation and eliminating persisters. This distinctive dual‐target effect not only enhanced its antibacterial efficacy but also served to mitigate potential risks associated with the development of drug resistance. In vivo experiments substantiated the efficacy of SR25 hydrogel, showcasing its capacity for antibacterial activity and the expedited healing of infected diabetic wounds. Consequently, this research offered a promising and optional strategy for the development of innovative antibacterial agents, specifically designed to address the complexities posed by challenging infections.

## 4 Experimental Section

### Bacteria Strains and Culture Conditions

The strain DL99^T^ was isolated from the intestinal tract of dairy cow samples from Guangze Farm (125° 55′ 10.59′' N 44° 10′ 27.97′' E) in Jilin, China. The primary colonizers were detected on ISP2 agar during a 7‐day incubation period at 37 °C. The strain DL99^T^ was deposited in the China Center for Type Culture Collection (CCTCC) under the accession number CCTCC M20231421. Various bacterial strains were kept in laboratory, and *Listeria monocytogenes* EGD was a generous gift from Professor Chang‐Yong Cheng of Zhejiang A&F University. Every bacterial strain was cultured overnight at 37 °C and 180 rpm while being shaken in tryptic soy broth (TSB, Guangdong Huankai Microbial Sci.&Tech. Co., Ltd., China).

### Phylogenetic Analysis

The TIANGEN DNA Extraction Kit (Beijing, China) was used to isolate the genomic DNA in accordance with the manufacturer's instructions. Extracts were utilized for whole‐genome sequencing and 16S rRNA gene amplification, as previously reported.^[^ ^32^ ^]^ The 16S rRNA genes were subjected to phylogenetic analysis through maximum likelihood (ML) and the neighbor‐joining (NJ) method using Mega 11 software.^[^ ^33^ ^]^ Using 1000 replications of the bootstrap resampling approach, the topologies were evaluated.

### Morphological and Biochemical Characterization

ISP2 medium supplemented with NaCl (0.5–5%, w/v) was used to test the tolerance to NaCl, and tests were conducted on growth at various pH values (2.0–10.0) and temperature ranges (4–50 °C). The gram reaction and the bacterial biochemical identification were measured as described earlier.^[^ ^34^ ^]^

### Genome Sequencing and Analysis

At Novogene Bioinformatics Technology Co., Ltd. (Beijing, China), the entire genome of the strain DL99^T^ was sequenced utilizing the Illumina NovaSeq PE150 and the Nanopore PromethION platform. The anti‐SMASH database analyzed the secondary metabolism gene clusters.^[^ ^35^ ^]^ The ANI and dDDH values between the strain DL99^T^ and its phylogenetic neighbors were determined as described earlier.^[^ ^34^ ^]^

### Identification of Potential AMPs

Only open reading frames (ORFs) shorter than 200 base pairs (bps) were chosen from the DL99^T^ genome. For AMP assessment, guidelines included helical structure or disulfide bonds, positive net charge, protein binding potential > 0 kcal mol^−1^, and peptide formation rate > 0.9. Peptide properties were determined by APD3,^[^ ^36^ ^]^ CAMPR3,^[^ ^37^ ^]^ HydrAMP^[^ ^38^ ^]^ and ExPASy.^[^ ^39^ ^]^ I‐TASSER was used to establish the peptide structural models.^[^ ^40^ ^]^

### Synthesis and Characterization of Peptides

Peptides were synthesized at NJ Peptide Co., Ltd. (Nanjing, China) by Fmoc‐based solid‐phase synthesis. The ESI‐MS (Agilent‐6125b) was used to determine the molecular weights of the peptides. The purity and retention time of the peptides were measured by RP‐HPLC. The chromatographic column used was a Symmetrix ODS‐R (4.6 × 250 mm, 5 µm). A nonlinear water/acetonitrile gradient containing 0.1% trifluoroacetic acid was used for 25 min, and the flow rate was 1.0 mL min^−1^. CD spectrum at 25 °C (Bio‐Logic MOS‐500, France). The contents (%) of the α helix and β sheet were calculated by k2d3 (http://k2d3.ogic.ca).

### MIC Assay

The MICs of the peptides were determined using the microplate broth dilution method.^[^ ^32^ ^]^ The bacteria in the mid‐log phase were diluted to 5 × 10^5^ colony forming units (CFU) mL^−1^ in TSB. 100 µL of serially diluted peptide solution and 100 µL of diluted bacteria were put to a sterile 96‐well plate. With no bacterial growth, the MIC was at its lowest concentration following an 18 h incubation period at 37 °C.

### Cytotoxicity Assay

The CCK‐8 kit was utilized to evaluate the cytotoxicity of the peptides on RAW264.7 and L929 cell lines. After plating the cells at a density of 2 × 10^5^ cells per well, they were incubated at 37 °C with 5% CO_2_ for an entire night. After exposure to various peptide concentrations for 48 h, each well received CCK‐8 solution (Beyotime, Shanghai, China). Following a 1 h incubation at 37 °C, the absorbance was determined with a microplate reader (BioTek, USA). Cell viability was then calculated following the guidelines provided by the manufacturer.

### Hemolysis Assay

Hemolytic activity of peptides was assessed by measuring hemoglobin release from mouse red blood cells (RBCs).^[^ ^41^ ^]^ Fresh RBCs were diluted to 2% (v/v) and mixed with a two‐fold serial peptide solution dilution and allowed to incubate for 1 h at 37 °C. An optical density (OD) measuring device (BioTek microplate reader, set at 490 nm) was used to measure the supernatants.

### Antibacterial Kinetics

During the logarithmic growth phase, 1 × 10^5^ CFU mL^−1^ of *E. coli* O157:H7 was diluted in 1 mL, the peptides were incubated with the bacterial solution for several intervals.^[^ ^42^ ^]^ The solution was diluted to a suitable ratio and evenly distributed across the TSB agar plates. Before being counted, colonies were cultured for one night at 37 °C.

### Resistance Development Assay

The sequential passaging approach was used to test the potential of SR25.^[^ ^32^ ^]^ For inducing resistance in *E. coli* O157:H7, colistin and ampicillin served as controls. For inducing resistance in MRSA USA300, vancomycin and ciprofloxacin served as controls. The sub‐MIC strains were cultivated overnight in TSB until they reached the mid‐logarithmic phase. Subsequently, the culture was diluted to 1 × 10^5^ CFU mL^−1^ in order to determine the MIC assay, which was repeated for 20 passages.

### Antibiofilm Activity Assay

A crystal violet (CV; Solarbio, Beijing, China) staining experiment was used to assess the biofilm inhibitory of SR25 on *E. coli*. Bacteria (1 × 10^6^ CFU mL^−1^) were incubated with SR25 for 24 h at 37 °C. *E. coli* biofilm was cleaned twice with PBS, then immobilized in methyl alcohol for 15 min, dried at 25 °C, and stained for 15 min with 0.1% CV. The dissolved CV in 95% ethanol determined the biofilm mass (OD at 595 nm). For biofilm eradication, after incubating the bacterial suspension for 24 h, SR25 and colistin solutions were added for 24 h. The subsequent steps mirrored the biofilm inhibition assay.

### Effect of SR25 on *E. coli* Persisters

Previously recognized and widely used procedures for developing persisters. The bacterial cells, with a turbidity of 0.8 at 600 nm, were obtained in the mid‐log phase. The culture was cultured at 37 °C for 30 min after 100 µg mL^−1^ rifampicin (Macklin, Shanghai, China) was added. The rifampicin‐treated culture was centrifuged, and the pellet was resuspended in TSB containing ampicillin (final concentration: 300 µg mL^−1^; Macklin, Shanghai, China) to remove nonpersister cells. After washing and resuspending in PBS, different concentrations of SR25, colistin, and ampicillin (final concentration: 3,000 µg mL^−1^, which is sufficient to remove persisters) were added. Then cultured for 24 h, spotted on TSB agar, and incubated overnight at 37 °C.

### Stability Assays

The SR25 was incubated in water at various temperatures for various durations to determine its thermal stability assay. The SR25 was incubated for various durations at 37 °C with varying pH values of PBS (pH = 2.0 or 12.0) for the acid‐base stability tests. The MIC values were subsequently identified at pH 6.0. To investigate the protease sensitivity of SR25 and melittin. Briefly, 10 m_M_ PBS (for pepsin pH = 2.0, for trypsin pH = 8.0) was used to generate a solution with a 20:1 or 100:1 peptide/protease molar ratio which was then incubated for 1 h at 37 °C. The samples underwent a 30 min boil. The mixture was centrifuged and the supernatant was collected after the proteases were precipitated for 30 min at 13,000 g. The MIC values were identified as mentioned above. A 50% trifluoroethanol (TFE) solution was used to dissolve each sample for the CD spectra.

### Membrane Permeability Assay

As previously described,^[^ ^43^ ^]^ the 1‐N‐phenylanphthylamine (NPN) uptake test was used to measure the OM permeability of SR25. Bacterial cells were collected and diluted in HEPES buffer. A 96‐well black plate was filled with 90 µL of peptide solution, 20 µL of the NPN solution (10 µmol L^−1^), and 90 µL of the bacterial suspension. The fluorescence was recorded (emission *λ* = 420 nm, excitation *λ* = 350 nm). The O‐nitrophenyl‐beta‐D‐galactopyranoside (ONPG) assay was used to evaluate the change in permeability of the bacterial IM.^[^ ^44^ ^]^ The bacterial suspension, along with 10 µL of ONPG (30 m_M_) and peptide solutions, was placed in a 96‐well plate. The OD at 420 nm was recorded every 10 min over 120 min.

### Leakage of Nucleic Acid and Protein

The treatment and untreated (control) cell suspensions were centrifuged to separate out the supernatants.^[^ ^32^ ^]^ The supernatants were collected, and determined the supernatant's absorbance at 260 or 280 nm to monitor protein or nucleic acid leakage, respectively.

### Scanning Electron Microscopy

SEM was utilized to track the morphological modifications in *E. coli* after treatment with SR25. The *E. coli* cells, adjusted to ≈10^5^ CFU mL^−1^, were exposed to SR25 at 1 × MIC. Following collection, 0.25% glutaraldehyde was used to fix the cells for an entire night, dehydrated using ethanol, and subjected to vacuum freeze–drying. Prior to examination, the samples were subjected to gold sputter coating and evaluated at 3 kV using a Nova NanoSEM650 scanning electron microscope (Frequency Electronics, Inc., USA).

### Intracellular ATP and Reactive Oxygen Species Assay

The intracellular ATP levels were measured using an Enhanced ATP Assay Kit (Beyotime, Shanghai, China). Bacterial cell overnight cultures were treated with SR25 at different doses for 1 h at 37 °C. The cultures were then gathered, centrifuged, and preserved in suspension. As directed by the manufacturer, the intracellular ATP concentrations were determined and computed using the standard curve. The levels of ROS were measured in compliance with the manufacturer's instructions using a ROS assay kit (Beyotime, Shanghai, China).

### Transcriptomics and Quantitative Real‐time Polymerase Chain Reaction (qRT–PCR) Analysis

Three replicates of *E. coli* were incubated overnight for 1 h at 37 °C with or without SR25 (1 × MIC). The total RNA of the bacteria was extracted using an RNAprep Pure Cell/Bacteria Kit (TIANGEN, Beijing, China). Every RNA sample was examined using an Agilent 5300 Bioanalyzer (Agilent Technologies, Beijing, China) to ensure its quality. A SuperScript double‐stranded cDNA synthesis kit (Invitrogen, CA) was used to generate double‐stranded cDNA, submitted for sequencing on an Illumina HiSeq 6000 at Shanghai Majorbio Biopharm Technology Co., Ltd. (China). The DEGs were determined by calculating the fold change (log_2_FoldChange > 1) and *p*‐value (< 0.05).

Using Power SYBR Green PCR Master Mix (Applied Biosystems, USA) and the ABI QuantStudio 3 sequence detection system (Applied Biosystems, USA), the expression of selected DEGs was verified by the qRT‐PCR experiment. The comparative critical threshold value (2^−ΔΔCT^) approach was applied to evaluate the changes in transcription levels, as described earlier.^[^ ^45^ ^]^ Table S8 (Supporting Information) includes a list of primers for the chosen DEGs in this study.

### Metabolomics Analysis

*E. coli* overnight cultures were coincubated for 1 h at 37 °C with or without SR25 (1 × MIC). Following a 10 min, 12 000 rpm centrifugation, the cells were cleaned in PBS and crushed in liquid nitrogen. The suspension received 80% v/v methanol, and after centrifugation, the diluted supernatant was injected into the liquid chromatography‐tandem mass spectrometry system at a final concentration of 50%. Metabolite characteristics were screened and analyzed using the internal standard normalization procedure. The R package MetaX processed the 3D data. MetaboAnalyst (http://www.metaboanalyst.ca/) and KEGG (Kyoto Encyclopedia of Genes and Genomes, http://www.kegg.jp) were utilized enrichment analysis.

### Measurement of Enzyme Activity

Overnight cultures of bacteria were collected to isolate the membranes according to the kit's introductions. After 2 min of incubation with SR25, the SDH and SQR activities were determined and calculated with a Succinate Dehydrogenase Kit and a Succinate‐quinone Reductase Kit (Shanghai Enzyme‐linked Biotechnology Co., Ltd., Shanghai, China).

### Molecular Docking and Molecular Dynamics Simulations

The typical docking procedures of SR25 and SQR (PDB code: 2WDV) were carried out using ZDock.^[^ ^46^ ^]^ Gromacs2022.3 software handled the simulations and trajectory analysis.^[^ ^47^ ^]^ The Amber99sb‐ildn force field and TIP3P water model were used in these simulations. The Molecular Mechanics Poisson‐Boltzmann Surface Area (MM/PBSA) approach was used to analyze the binding free energy between the SQR and SR25. To analyze the trajectories, the analysis tools PyMOL, Gromacs, and VMD were applied. The Discovery Studio Platform's Calculate Mutation Energy (Binding) module performed the computational alanine scanning, replacing each amino acid in the SQR with alanine and calculating energy differences between the wild type and mutants.

### Construction, Expression and Purification of SdhD and its Mutants

After being extracted from *E. coli*, the *sdhD* gene was cloned and inserted into the expression vector pET‐28a(+) and digested using the endonucleases *Xho* I and *Bam*H I. Using a QuikChange site‐directed mutagenesis kit (Stratagene, USA), the expression vectors for the SdhD mutants were constructed, and Table S9 (Supporting Information) lists the gene‐specific primers used. A prokaryotic expression system was used for the purification of SdhD and its mutants.

### Fluorescence Quenching Analysis

Using an RF‐5301 PC fluorescence spectrometer (Shimadzu, Japan), fluorescence quenching studies were conducted. The fluorescence intensities of SdhD and its mutants were titrated with increasing SR25 concentrations in PBS. The excitation wavelength was set at 280 nm, and the emission spectra were recorded between 300 and 390 nm. The quenching of fluorescence was characterized according to the linear Stern–Volmer equation.^[^ ^48^ ^]^

### Preparation of Hydrogels

Sodium alginate (SA) (1% w/v) was added to the SR25 solution (50 µg mL^−1^), pre‐swelled for 1 h, and stirred for 30 min. Simultaneously, 1% w/v gelatin was added to deionized water, pre‐swelled for 40 min, and stirred for 30 min at 60 °C. All solutions were bubble‐free, achieved using an ultrasonic cleaner. Then, CaCO_3_ (0.3% w/v) was sonicated in a gelatin solution. After combining the gelatin solution and SA/SR25 solution and stirring for 5 min, gluconolactone (0.75% w/v) was added and vigorously agitated. An ultrasonic cleaner was then used to eliminate any remaining air bubbles. To generate a hydrogel, the mixture was then put into a mold and left at 4 °C overnight.

### Antimicrobial Activity Assay of CaAGEAM Hydrogel in vitro

The CaAGEAM hydrogel's antibacterial efficacy against MRSA and *E. coli* was evaluated using the inhibition zone method. The combined bacterial sample (1 × 10^8^ CFU mL^−1^) was applied dropwise in 100 µL to a TSB agar plate, and then the plate was evenly coated with a coating bar. Placed on the agar plate, the eight‐mm diameter hydrogel was incubated at 37 °C and swapped out every 12 h on a fresh plate coated with bacteria. A digital camera was used to measure and record the inhibitory zone's diameter.

### Basic Properties of CaAGEAM Hydrogel

The release of SR25 from the CaAGEAM hydrogel was assessed in PBS. 20 mL of PBS and 1 mL of hydrogel were combined and shook constantly. Fresh media (1 mL) replaced the collected release media at different intervals, and the concentration of released AMP was measured at 220 nm.

Gravimetry was used to determine the degree of swelling of the CaAGEAM hydrogel. The 0.8 mm diameter dried hydrogel was initially weighed, submerged in 20 mL of PBS at 25 °C for 24 h. The hydrogel was periodically removed from the PBS, the excess buffer was removed with filter paper, and the mixture was weighed. The swelling percentage and water content were calculated as described previously.^[^ ^49^ ^]^ To assess water retention, the dry hydrogel sample was weighed and then exposed to room temperature. Changes in weight were recorded over time.

### Cytocompatibility Assay of CaAGEAM Hydrogel

The CaAGEAM hydrogel was placed on 96‐well plates. After 3 h of sterile UV radiation, the samples underwent three PBS washes. The next procedures were performed in a manner similar to that used for the cytotoxicity and hemolysis assays.

### Treatment of Diabetic Mouse Wounds

The male C57BL/6J mice were obtained from Liaoning Changsheng Technology Industrial Co., Ltd. and weighed roughly 20 g. In order to establish animal models of diabetes, streptozotocin (STZ) (50 mg kg^−1^) was injected intraperitoneally once daily for five days in a citrate buffer solution (0.1 mol L^−1^, pH 4.5). Blood glucose levels were monitored using a commercial glucometer, and successful induction of hyperglycemia was confirmed when fasting blood glucose reached 16.7 mmol L^−1^. Forty mice were randomly divided into four groups: PBS, CaAGE, KFX (Kangfuxin; Haoyisheng, Sichuan, China), and CaAGEAM. Sodium pentobarbital (0.3%/100 g) was injected intraperitoneally to anaesthetize the mice, and full‐thickness skin wounds were created using an 8 mm biopsy punch. For each wound, 20 µL of a mixed bacterial suspension was inoculated (10 µL of 1 × 10^8^ CFU mL^−1^ *E. coli* and 10 µL of 1 × 10^8^ CFU mL^−1^ MRSA). Treatment began one day after the bacterial challenge and was administered once every two days. Daily measurements and digital pictures of the wound area were taken to monitor the healing process.

### Antimicrobial Activity Assay of CaAGEAM Hydrogel In Vivo

The removed skin tissue was homogenized in PBS on day 3. The homogenates were then plated on selection agar plates after being serially diluted. Selective cultures of *E. coli* and MRSA were performed using eosin‐methylene blue agar and mannitol salt agar, respectively.

### Histology Analysis

After cleaning with PBS, the ulcerated tissues or primary organs were preserved in 4% (w/v) paraformaldehyde and embedded in paraffin.^[^ ^50^ ^]^ The tissues were serially sectioned at 5 µm. Following the manufacturer's directions, sections were collected and stained with MT and H&E. The MT staining images were analyzed using ImageJ software, and the collagen fibers in the blue channel were examined.

### Statistical Analysis

The means ± standard deviations of independent runs were used to show all the data. To perform the statistical analysis, GraphPad Prism 9.0 was used. Unpaired Student's *t*‐tests or one‐way analysis of variance were used for group comparisons. *p* values that were less than 0.05 were regarded as statistically significant. (^*^ *p* < 0.05, ^**^ *p* < 0.01, ^***^ *p* < 0.001, ^****^ *p* < 0.0001).

### Ethics Declarations

All in vivo animal experiments were conducted according to the institutional guidelines and relevant regulations for Animal Experimentation of Laboratory Animals of Jilin University, and all animal experiments were approved by the Jilin University Animal Center's Ethics Committee (KT201902111).

## Conflict of Interest

The authors declare no competing interests.

## Supporting information

Supporting Information

Supporting Information

## Data Availability

The data that support the findings of this study are available from the corresponding author upon reasonable request.
