# Supplementary material for: Dual‐Mechanism Peptide SR25 has Broad Antimicrobial Activity and Potential Application for Healing Bacteria‐infected Diabetic Wounds
Source: Adv Sci (Weinh). 2024 Jun 14;11(30):2401793. doi: 10.1002/advs.202401793 (PMC11321617; doi:10.1002/advs.202401793)
Supplement: Supplementary file 1 — Supporting Information [file ADVS-11-2401793-s001.doc]

**Supporting Information**

**Dual-mechanism Peptide SR25 has Broad Antimicrobial Activity and Potential Application for Healing Bacteria-infected Diabetic Wounds**

*Xue-Yue Luo*^†^*, Chun-Mei Hu*^†^*, Qi Yin*^†^, *Xiao-Mei Zhang, Zhen-Zhen Liu, Cheng-Kai Zhou, Jian-Gang Zhang, Wei Chen*, Yong-Jun Yang**

LXY, HCM, YQ, ZXM, LZZ, ZCK, ZJG, CW, YYJ

Department of Preventive Veterinary Medicine, College of Veterinary Medicine, Jilin University, Changchun Jilin Province, 130062 P. R. China

^†^ These authors contributed equally to this work.

*** Correspondence to Yong-Jun Yang ([youngjune@jlu.edu.cn](mailto:youngjune@jlu.edu.cn)); Wei Chen (chw_cc@jlu.edu.cn).

**Table S1.** Biochemical features that differentiate strain DL99^T^ and its closest phylogenetic neighbours strain.

| **Characteristic** | ***Nonomuraea Jilinensis* DL99^Ta,b^** | ***Nonomuraea fastidiosa* 104^T^** |
| --- | --- | --- |
| ONPG test | + | + |
| Arginine dihydrolase | + | + |
| Lysine decarboxylase | + | + |
| Ornithine decarboxylase | + | + |
| Utilization of citrate | + | + |
| H_2_S production | − | − |
| Hydrolysis of gelatin | − | + |
| Cellulolytic test | − | − |
| Utilization of glucose | + | + |
| Utilization of sucrose | − | − |
| Splitting of urea | + | + |
| Milk coagulation | − | ND^c^ |
| pH optimum (range) | 7.0 (4.0–10.0) | ND |
| NaCl (%w/v) | 0–5.0 | 0–2.5 |

^a)^ +: Positive (growth or reaction).

^b)^ −: Negative (no growth or no reaction).

^c)^ No data available.

**Table S2.** ANI and dDDH between strain DL99^T^ and other species of Nonomuraea.

|  |  | **DL99^T^** | | |
| --- | --- | --- | --- | --- |
| **Strain** | **Reference genome** | **ANIm^a^** | **ANIb^b^** | **dDDH** |
| *Nonomuraea Africana* DSM 43748^T^ | GCA_014873535.1 | 84.89 | 77.48 | 21.37±1.46 |
| *Nonomuraea angiospora* DSM 43173^T^ | GCA_014873145.1 | 86.39 | 82.34 | 27.07±0.78 |
| *Nonomuraea aridisoli* KC333^T^ | GCA_003236395.1 | 86.92 | 82.83 | 29.37±0.9 |
| *Nonomuraea candida* NRRL B-24552^T^ | GCA_000725485.1 | 87.48 | 84.07 | 30.67±0.81 |
| *Nonomuraea cavernae* CGMCC 4.7368^T^ | GCA_014646355.1 | 85.44 | 79.64 | 24.5±0.85 |
| *Nonomuraea coxensis* DSM 45129^T^ | GCA_000379885.1 | 86.39 | 81.83 | 29.03±1.95 |
| *Nonomuraea cypriaca* K274^T^ | GCA_015645445.1 | 86.18 | 81.92 | 26.63±0.86 |
| *Nonomuraea deserti* KC310^T^ | GCA_004348685.1 | 86.39 | 82.29 | 28.23±0.84 |
| *Nonomuraea diastatica* KC712^T^ | GCA_004349015.1 | 86.24 | 82.13 | 27.53±0.65 |
| *Nonomuraea dietziae* DSM 44320^T^ | GCA_014195505.1 | 84.73 | 77.35 | 21.2±1.42 |
| *Nonomuraea endophytica* DSM 45385^T^ | GCA_014203235.1 | 84.9 | 77.74 | 21.3±1.84 |
| *Nonomuraea fuscirosea* CGMCC 4.7104^T^ | GCA_003001935.1 | 87.07 | 83.92 | 30.37±1.22 |
| *Nonomuraea glycinis* CGMCC 4.7430^T^ | GCA_014646515.1 | 85.25 | 79.33 | 24.27±0.67 |
| *Nonomuraea harbinensis* CGMCC 4.7106^T^ | GCA_019396385.1 | 85.42 | 79.61 | 25.2±0.78 |
| *Nonomuraea indica* DRQ-2^T^ | GCA_002850745.1 | 85.69 | 79.49 | 24.4±1 |
| *Nonomuraea jabiensis* DSM 45507^T^ | GCA_014204795.1 | 86.48 | 82.3 | 26.8±1 |
| *Nonomuraea jiangxiensis* CGMCC 4.6533^T^ | GCA_900099965.1 | 86.12 | 81.77 | 26.67±0.67 |
| *Nonomuraea lactucae* NEAU-YG30^T^ | GCA_003313395.1 | 85.74 | 79.39 | 23.23±2.16 |
| *Nonomuraea longispora* KC201^T^ | GCA_004348345.1 | 86.23 | 81.84 | 28.57±1.22 |
| *Nonomuraea maritima* CGMCC 4.5681^T^ | GCA_900100395.1 | 86.39 | 81.8 | 28.83±1.37 |
| *Nonomuraea mesophile* 6K102^T^ | GCA_004352805.1 | 86.26 | 82.02 | 28.73±1.46 |
| *Nonomuraea montanisoli* SMC 257^T^ | GCA_013363975.1 | 85.7 | 79.41 | 23.83±1.11 |
| *Nonomuraea muscovyensis* DSM 45913^T^ | GCA_014207745.1 | 85.63 | 79.47 | 24.23±0.9 |
| *Nonomuraea phyllanthi* PA1-10^T^ | GCA_006334985.2 | 86.51 | 82.39 | 28.4±0.95 |
| *Nonomuraea polychrome* DSM 43925^T^ | GCA_004011505.1 | 86.24 | 81.86 | 26.47±0.78 |
| *Nonomuraea pusilla* DSM 43357^T^ | GCA_900109355.1 | 86.05 | 80.44 | 25.93±0.64 |
| *Nonomuraea rhizosphaerae* CGMCC 4.7431^T^ | GCA_019396405.1 | 85.65 | 79.23 | 24.27±1.1 |
| *Nonomuraea rubra* DSM 43768^T^ | GCA_014207985.1 | 87.26 | 84.09 | 30±0.72 |
| *Nonomuraea solani* CGMCC 4.7037^T^ | GCA_900108335.1 | 86.55 | 82.95 | 27.67±0.76 |
| *Nonomuraea soli* DSM 45533^T^ | GCA_013761175.1 | 84.47 | 76.42 | 20.23±1.31 |
| *Nonomuraea spiralis* JCM 3286^T^ | GCA_014648435.1 | 86.03 | 79.66 | 25.93±0.74 |
| *Nonomuraea terrae* CH32^T^ | GCA_004348995.1 | 86.8 | 81.86 | 29.47±1.1 |
| *Nonomuraea turkmeniaca* DSM 43926^T^ | GCA_005889735.1 | 86.18 | 80.33 | 26..3±1 |
| *Nonomuraea wenchangensis* CGMCC 4.5598^T^ | GCA_900111685.1 | 86.31 | 81.05 | 28.7±1.75 |
| *Nonomuraea zeae* DSM 100528^T^ | GCA_005889725.1 | 86.34 | 79.94 | 26.83±0.86 |

^a)^ average nucleotide identity based on MUMmer.

^b)^ average nucleotide identity based on BLAST.

**Table S3. The sequence comparison of the three antimicrobial peptides with those in the databases.**

| **Peptide**  **Database** | **Similarity percentage in APD3 (%) ^a)^** | **Sequence number in APD3** | **Score in CAMPR3 ^b)^** | **Sequence number in CAMPR3** |
| --- | --- | --- | --- | --- |
| SR25 | 42.31 | AP04208 | 20.4 | CAMPSQ2032 |
| RA25 | 44.44 | AP01633 | 21.2 | CAMPSQ1588 |
| FR24 | 42.31 | AP04208 | 23.1 | CAMPSQ388 |

^a)^ The data is the highest similarity percentage compared to sequences in the APD3 database.

^b)^ The data is the highest score compared to sequences in the CAMPR3 database.

**Table S4.** KEGG Pathway enrichment analysis of differentially expressed genes.

Refer the attached Excel spreadsheet.

**Table S5.** KEGG Pathway enrichment analysis of integrated transcriptomics and metabolomics data.

Refer the attached Excel spreadsheet.

**Table S6.** Residues of the interaction between SR25 and SQR.

| **SR25 Residue** | **SQR Residue** | **Distance (Å)** | **H Binding** | **Salt Bridge** | **Pi Stacking** |
| --- | --- | --- | --- | --- | --- |
| Arg22 | D: Ile70 | 1.7 |  |  |  |
| Arg22 | D: Ile74 | 1.8 |  |  |  |
| Arg19 | C: Asn237 | 2 | 2 |  |  |
| Arg15 | D: Asp15 | 1.9 | 1 | 1 |  |
| Arg15 | D: Trp77 | 2.1 |  |  |  |
| Trp14 | D: Leu69 | 2 | 1 |  |  |
| Trp14 | D: Trp73 | 2.3 |  |  | 1 |
| Arg13 | D: Leu101 | 1.7 |  |  |  |
| Arg13 | D: Ile68 | 2.1 | 2 |  |  |
| Gly3 | D: Hie14 | 2.2 | 1 |  |  |
| Ala2 | D: Asp15 | 2 | 1 |  |  |
| Ser1 | D: Asp15 | 1.8 | 1 |  |  |
| Ser1 | D: Hie14 | 1.7 | 1 |  |  |

**Table S7.** Binding free energy analysis between SR25 and SQR.

| **Energy terms^a^** | **Mean (kcal mol^-1^)** |
| --- | --- |
| △*E_vdw_* | -185.98±8.36 |
| △*E_ele_* | -1923.34±9.45 |
| △*G_pol_* | 2008.96±6.67 |
| △*G_nonp_* | -26.30±0.33 |
| △*G_gas_* | -2109.31±12.62 |
| △*G_solv_* | 1982.66±6.68 |
| △*G_binding_* | -126.66±14.28 |

^a)^ *E_vdw_* van der Waals energy, *E_ele_* electrostatic energy, *G_pol_* polar solvation energy, *G_nonp_* nonpolar solvation energy, *G_gas_* gas phase energy, *G_solv_* solvation free energy.

**Tabel S8.** Primer sequences in qRT-PCR analysis.

| **Gene** | **Forward (5’ – 3’)** | **Reverse (5’** – **3’)** |
| --- | --- | --- |
| 16S rRNA | GGTGGGCATTAAAGGCGATT | AACCACTGGATTACGCTCCA |
| *fadB* | ATGATCCCGATGGTCAACGA | GTGCTGATATTGCTGTGCCA |
| *betB* | GTCGGGCCGTTGATATTCTG | ACAAAGGACGTTTCACGCAA |
| *frmA* | CGTCCATTCCAGTTGGTCAC | TATGCGTGACAAACGGTTCC |
| *sdhD* | TCGTCCTGACGCTCTACATC | AACGTAGTCGGTCAACACCT |
| *sdhA* | TTGAGTGCCTGGAACTGGAT | CAGATACAGGGAGTGGCACA |
| *sdhB* | GATCCGCGATTTGGTGGTAG | ATTCATACAGCCCGTCGAGT |
| *sdhC* | GGGCAGCTTCTTCGTCAAAT | CTCCTGCGAGAAGTGAAAGC |
| *gntK* | TTCATGCCGCGTTTCTTGAT | AGAACAGACGATCAGCGACA |
| *cyoC* | ACCTATGCCGTTCTGGTGAA | ACCAAACAACCAGGTCAACG |
| *mdh* | GGTATGGATCGTTCCGACCT | AGCAATTGCAACTGTGGTGT |
| *pfkA* | AGCCGTGAAGACCTGGTAAA | GCCAGTTCGTCAACATCACA |
| *glxK* | TTTCGACCCTCTTTCCCGAT | CATCTCAATCACCGCCGTTT |
| *ilvM* | GTATCGGCTCGCTTCAATCC | CTGGCAACGGTCAATTCGAT |
| *malX* | CGCTGGAATTCCTGTTCCTG | CTGGCACCATGTACCACTTG |
| *gadB* | GGTTATCTGGCGTGACGAAG | TAAGCGGCAACCTGGTAAGA |
| *ttdA* | AGTTCAGTGATGGGCGTACA | CTCCGGGTGTGAGACAGATT |

**Table S9.** Primer sequences used for SDHD subunit purification and site-directed mutagenesis.

| **Primer** | **Sequence (5’ - 3’)^a, b^** |
| --- | --- |
| SDHD-WT F | CTG**GGATC**ATGGTAAGCAACGCCTCCGC |
| SDHD-WT R | CTG**CTCGA**TCACACACCCCACACCACAAC |
| SDHD-H14A F | GACGCAATGGCGTAGCGGATTTCATCCTCG |
| SDHD-H14A R | CGAGGATGAAATCCGCTACGCCATTGCGTC |
| SDHD-D15A F | GGCGTACATGATTTCATCCTCGTTC |
| SDHD-D15A R | GTTACCGCATGTACTAAAGTAGGAG |
| SDHD-L18A F | CATGATTTCATCGCGGTTCGCGCTAC |
| SDHD-L18A R | GGTAGCGCGAACCGCGATGAAATCATG |
| SDHD-F66A F | CTGCTGGCGCTGGCGTCTATCTTGATCC |
| SDHD-F66A R | GGATCAAGATAGACGCCAGCGCCAGCAG |
| SDHD-L69A F | GCTGTTTTCTATCGCGATCCATGCCTGG |
| SDHD-L69A R | CCAGGCATGGATCGCGATAGAAAACAG |
| SDHD-I70A F | GTTTTCTATCTTGGCGCATGCCTGGAT |
| SDHD-I70A R | GTTTTCTATCTTGGCGCATGCCTGGAT |
| SDHD-V98A F | CAACTGGTGATTGCGGTTGCACTGGTG |
| SDHD-V98A R | CACCAGTGCAACCGCAATCACCAGTTG |

^a)^ Restriction enzyme sites are underlined and bold.

^b)^ The mutated codons are underlined.


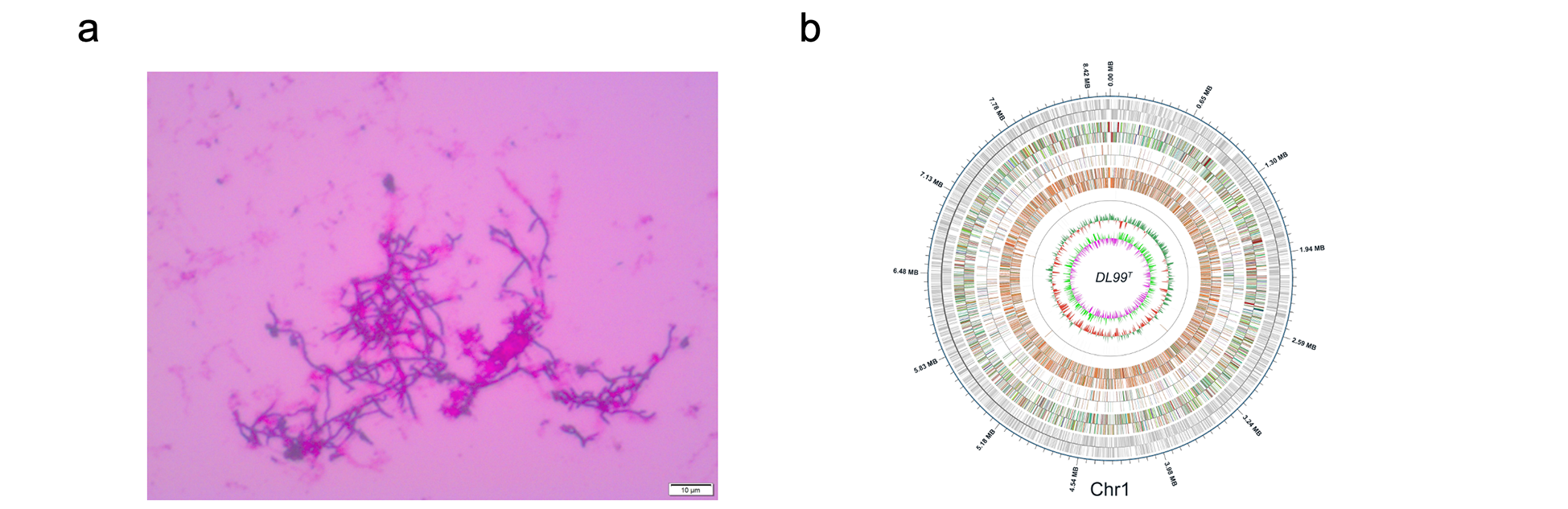


**Figure S1.** Morphological and genomic characteristics of *Nonomuraea Jilinensis* DL99^T^. a) Gram staining morphological observation of strain DL99^T^. Scale bar = 20 μm. b) Circular map for the whole genome of strain DL99^T^. From the outside to the center: genome sequence coordinates, gene annotation (COG, eggNOG, KEGG, and GO categories), ncRNA, GC content, and GC skew (G–C/G+C).


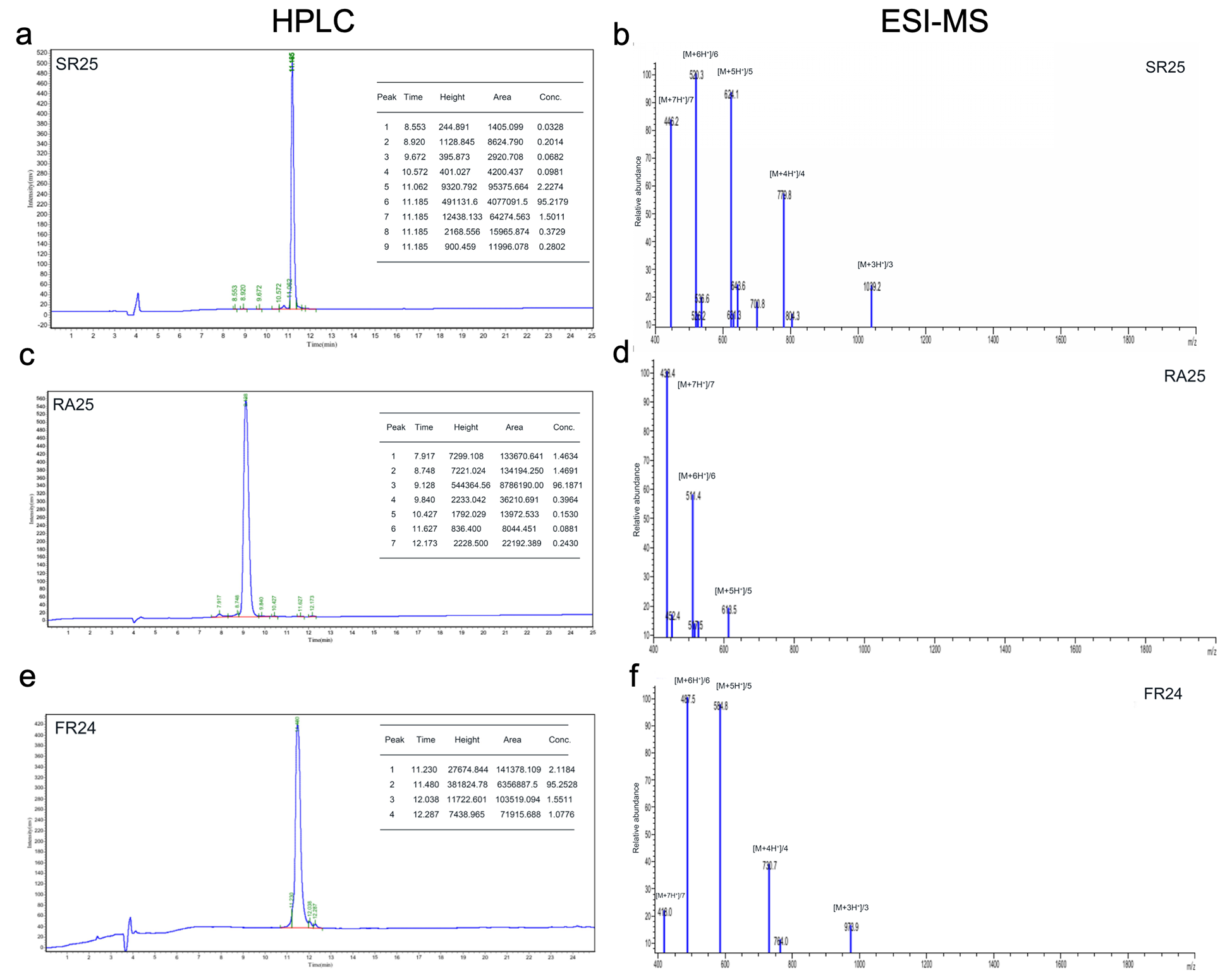


**Figure S2.** HPLC and MS data for the antimicrobial peptides. a) HPLC spectra of SR25. b) ESI-MS of SR25. c) HPLC spectra of RA25. d) ESI-MS of RA25. e) HPLC spectra of FR24. f) ESI-MS of FR24.


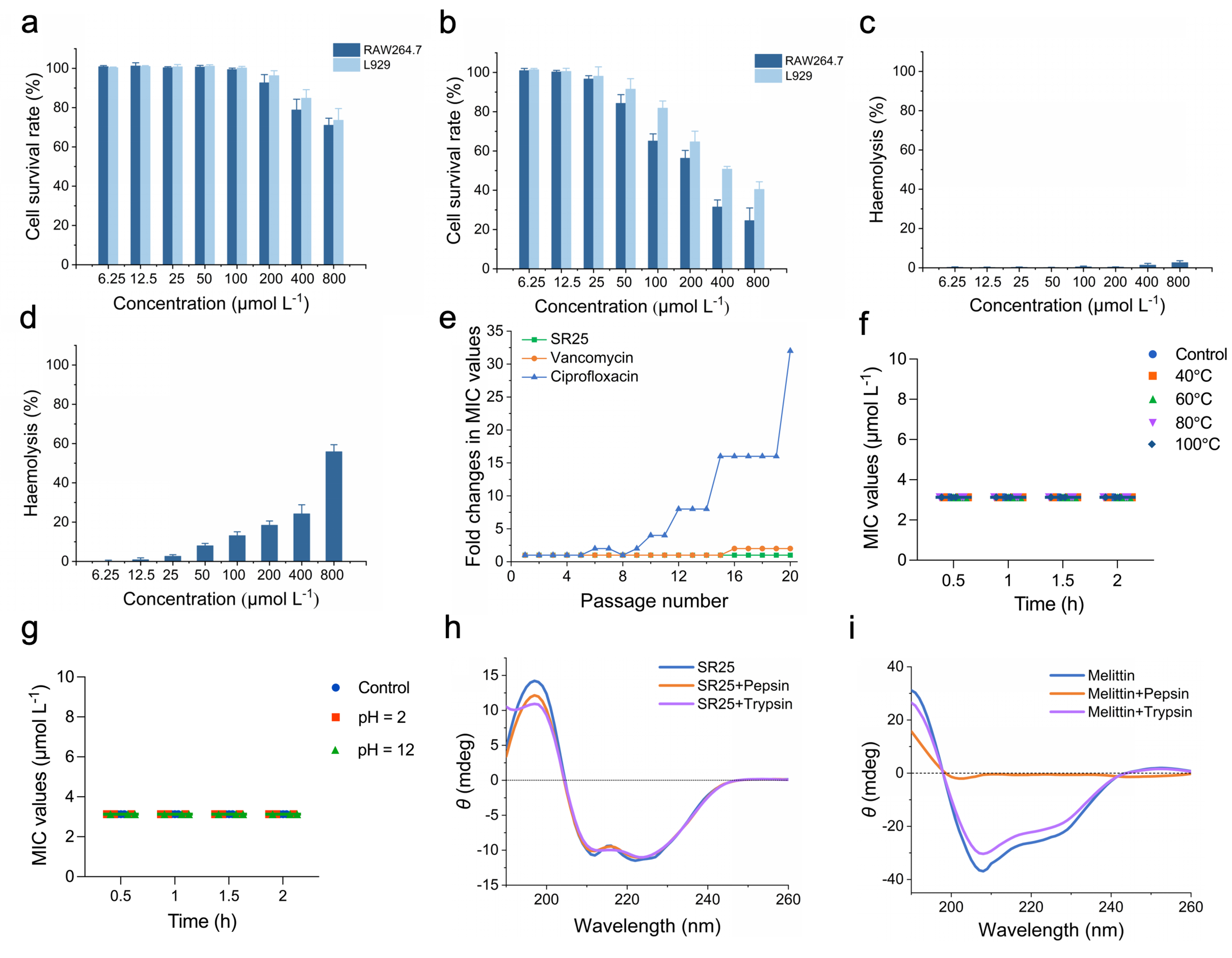


**Figure S3.** Cytotoxicity and stability of AMPs. a) Cytotoxicity of RA25 was evaluated using CCK-8 assay on Raw 264.7 cell line and L929 cell line. b) Cytotoxicity of FR24 was evaluated using CCK-8 assay on Raw 264.7 cell line and L929 cell line. c-d) Hemolytic activity of AMPs. Hemolytic activity of RA25 (c) and FR24 (d) to the red blood cells of C57BL/6 mice. 1% Triton X‐100 was used as a positive control, and PBS was used as a negative control. e) Induced resistance assay of SR25, vancomycin and ciprofloxacin against MRSA for a continuous passage of 20 cycles. f) Antibacterial stability of SR25 after treatment at different temperatures was evaluated by MIC values. g) Antibacterial stability of SR25 after treatment at extreme pH environments was evaluated by MIC values. h-i) Protease sensitivity of AMPs. CD spectra of SR25 (h) and melittin (i) treated with the proteases. The peptide concentrations were fixed at 100 μmol L^-1^. All of the samples were dissolved in 50% TFE. The peptide/protease molar ratio was 20:1.


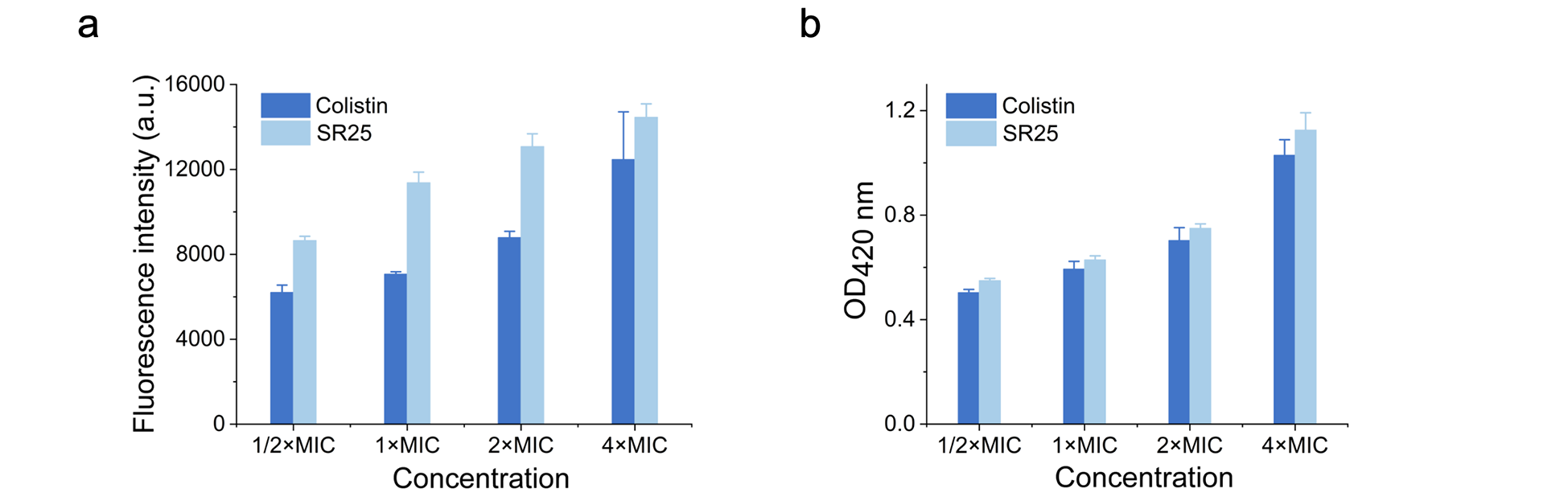


**Figure S4.** Effect of AMPs on membrane permeability. a) Outer membrane permeability of *E. coli* O157:H7 treated with different concentrations of SR25 and colistin for 1 h by NPN assay. b) Inner membrane permeability of *E. coli* O157:H7 treated with different concentrations of SR25 and colistin for 1 h indicated by the hydrolysis of ONPG.


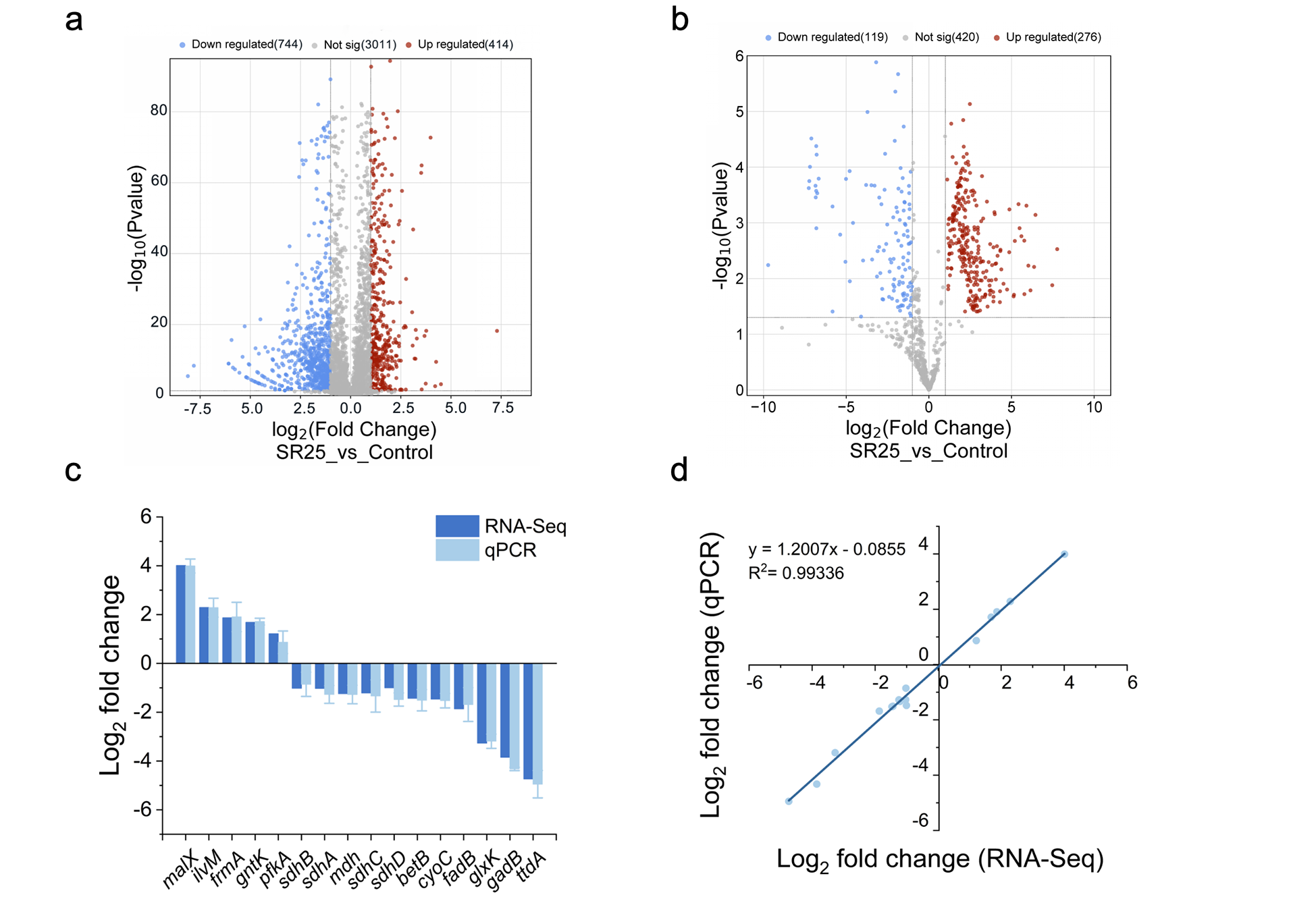


**Figure S5.** Transcriptomic and metabolomic analysis of *E. coli* treated with SR25. a) Volcano plots of DEGs. A total of 1158 DEGs, including 414 up-regulated and 744 down-regulated genes, threshold of significance as fold change was >2, FDR <0.05. b) Volcano plot of differential metabolites. Each point in the volcano plot represents a metabolite, and the horizontal coordinate represents the log value of the quantitative difference multiple of a certain metabolite in two samples. The vertical axis represents the VIP value. c) Validation of the RNA-Seq result by qPCR. The y-axis represents the log_2_ fold change of gene expression; x-axis shows the names of genes employed for validation. d) Correlation of RNA-Seq data and qPCR data on a log scale.


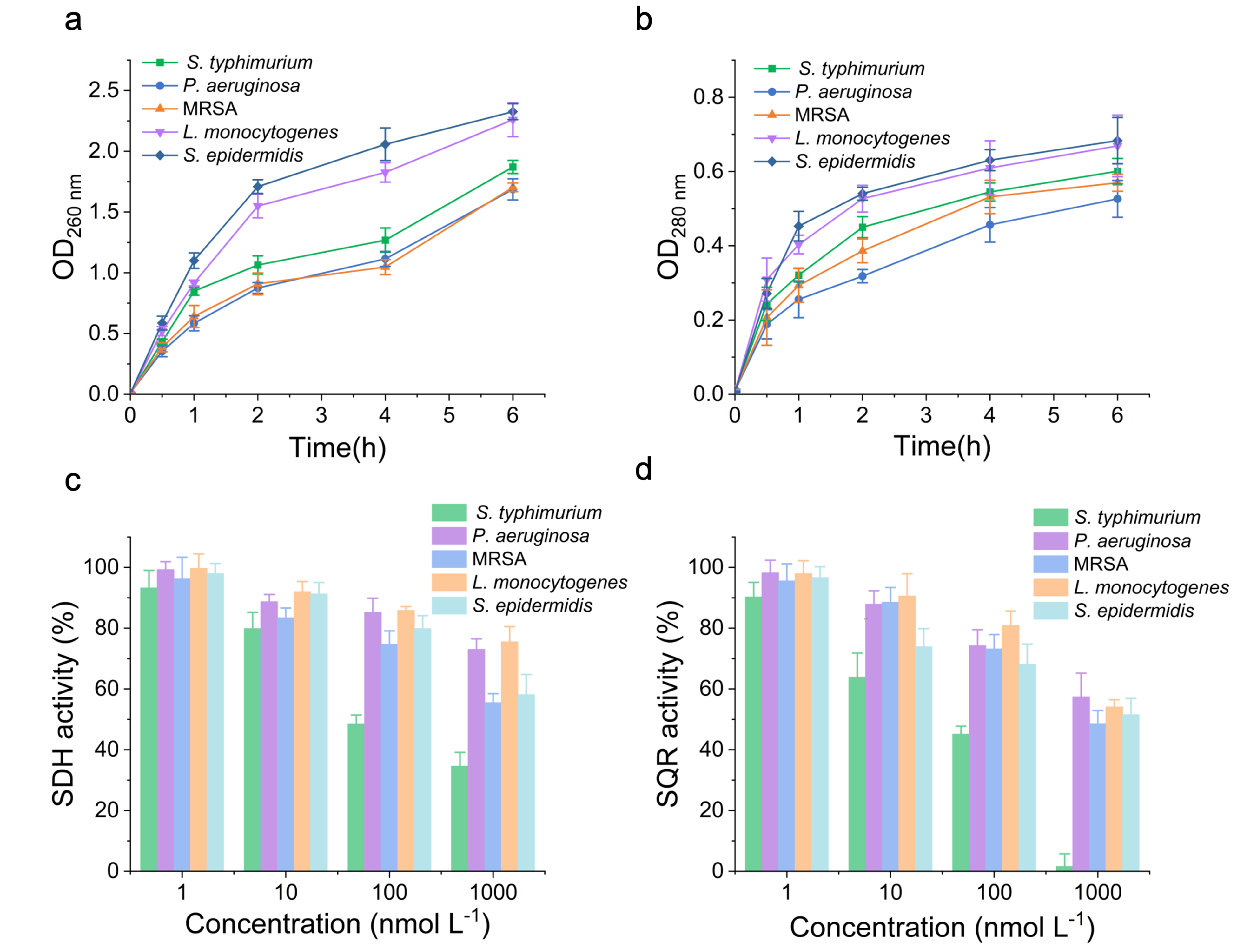


**Figure S6.** Effect of SR25 on membrane permeability and enzyme activity of other pathogens. a) Measurement of cellular leakage of nucleic acid after the treatment of SR25 (1 × MIC) for different durations (*n* = 3). b) Measurement of cellular leakage of protein after the treatment of SR25 (1 × MIC) for different durations (*n* = 3). c) Detection of SDH activity after SR25 treatment (*n* = 3). c) Detection of SQR activity after SR25 treatment (*n* = 3). MRSA, methicillin-resistant *Staphylococcus aureus*.


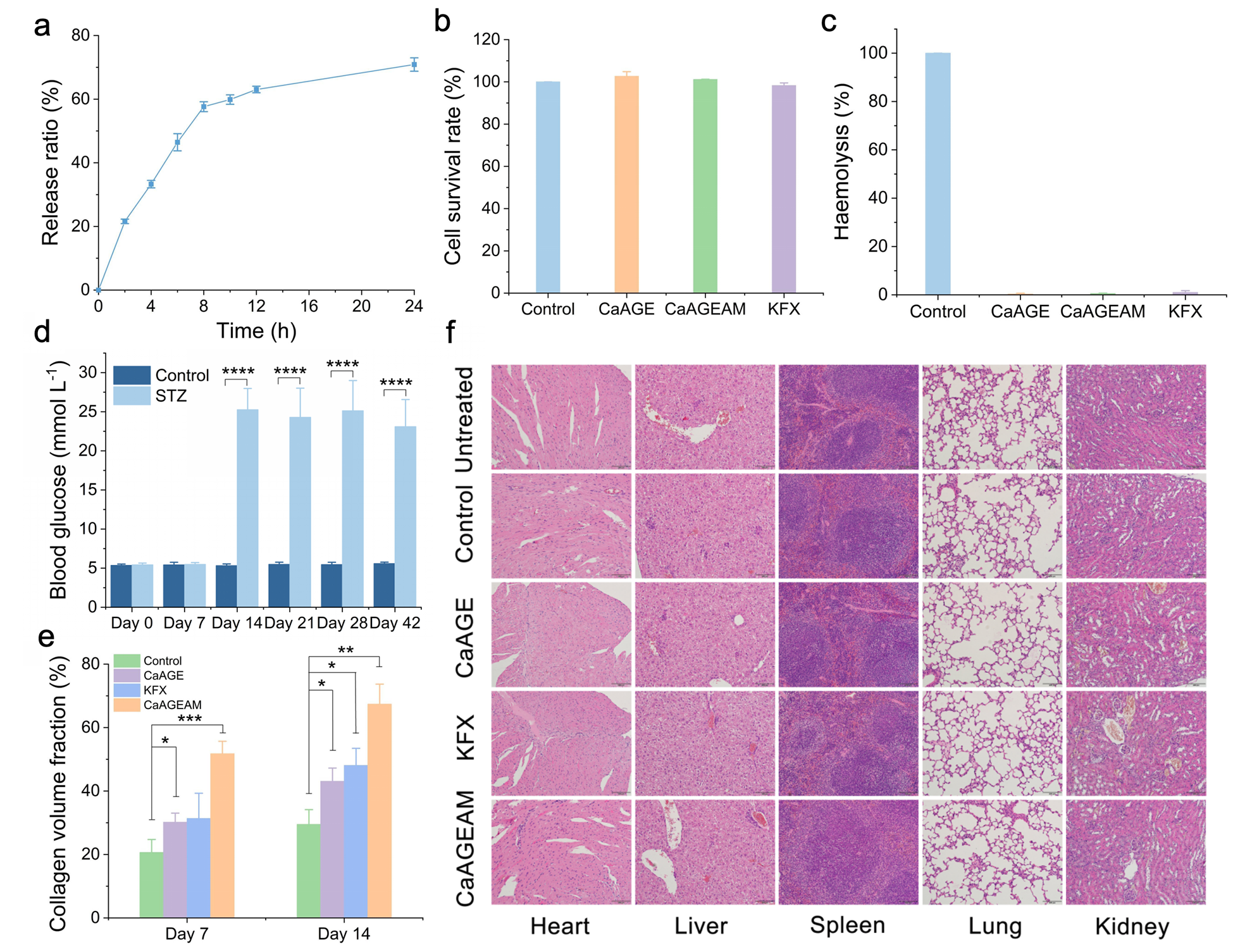


**Figure S7.** Application of SR25 hydrogel in wound infection model of diabetes mice. a) Release profile of antimicrobial peptide SR25 in CaAGEAM. b) Cell survival rate after 48 h incubation of CaAGEAM with L929 by CCK-8 assay. c) Hemolytic activity of hydrogel to the red blood cells of C57BL/6 mice. d) Blood glucose levels in diabetic mice throughout the wound healing periods. e) Quantitative calculation of relative collagen density by Image J. f) H&E staining of various organs from infected mice at the end of treatment. Scale bar = 100 μm. KFX represents Kangfuxin, CaAGE represents sodium alginate/Ca^2+^/gelatin gels, CaAGEAM represents sodium alginate/Ca^2+^/gelatin/antimicrobial peptide SR25. **p* < 0.05, ***p* < 0.01, ****p* < 0.001, *****p* < 0.0001.
